# Shift of large-scale atmospheric systems over Europe during late MIS 3 and implications for Modern Human dispersal

**DOI:** 10.1038/s41598-017-06285-x

**Published:** 2017-07-19

**Authors:** Igor Obreht, Ulrich Hambach, Daniel Veres, Christian Zeeden, Janina Bösken, Thomas Stevens, Slobodan B. Marković, Nicole Klasen, Dominik Brill, Christoph Burow, Frank Lehmkuhl

**Affiliations:** 1 Department of Geography, RWTH Aachen University, Templergraben 55, 52056 Aachen, Germany; 2 BayCEER & Chair of Geomorphology, University of Bayreuth, 94450 Bayreuth, Germany; 3 Laboratory for Paleoenvironmental Reconstruction, Faculty of Sciences, University of Novi Sad, Trg Dositeja Obradovića 2, 21000 Novi Sad, Serbia; 4 Romanian Academy, Institute of Speleology, Clinicilor 5, 400006 Cluj-Napoca, Romania; 5 Interdisciplinary Research Institute on Bio-Nano-Science of Babes-Bolyai University, Treboniu Laurean 42, 400271 Cluj-Napoca, Romania; 6 Department of Earth Sciences, Uppsala University, Villavägen 16, 75236 Uppsala, Sweden; 7 Institute of Geography, University of Cologne, Albertus-Magnus-Platz, 50923 Cologne, Germany

## Abstract

Understanding the past dynamics of large-scale atmospheric systems is crucial for our knowledge of the palaeoclimate conditions in Europe. Southeastern Europe currently lies at the border between Atlantic, Mediterranean, and continental climate zones. Past changes in the relative influence of associated atmospheric systems must have been recorded in the region’s palaeoarchives. By comparing high-resolution grain-size, environmental magnetic and geochemical data from two loess-palaeosol sequences in the Lower Danube Basin with other Eurasian palaeorecords, we reconstructed past climatic patterns over Southeastern Europe and the related interaction of the prevailing large-scale circulation modes over Europe, especially during late Marine Isotope Stage 3 (40,000–27,000 years ago). We demonstrate that during this time interval, the intensification of the Siberian High had a crucial influence on European climate causing the more continental conditions over major parts of Europe, and a southwards shift of the Westerlies. Such a climatic and environmental change, combined with the Campanian Ignimbrite/Y-5 volcanic eruption, may have driven the Anatomically Modern Human dispersal towards Central and Western Europe, pointing to a corridor over the Eastern European Plain as an important pathway in their dispersal.

## Introduction

**Figure 1:**
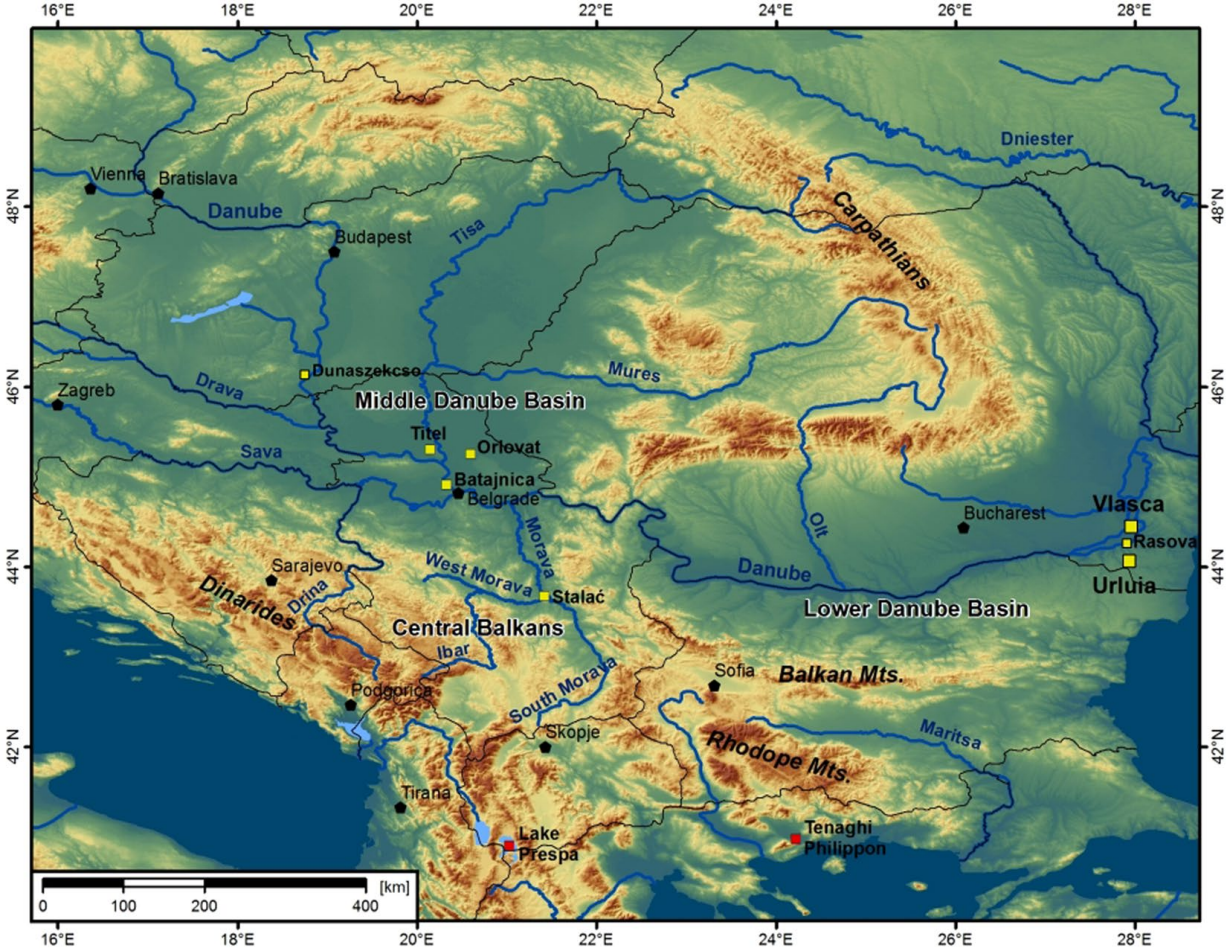
Map of the Southeastern Europe showing key loess-palaeosol sequences (Urluia (this study), Vlasca (this study), Rasova^12^, Titel^37, 40^, Batajnica^5, 11^, Orlovat^7^, Stalać^2^, Dunaszekcső^9^) and lacustrine records (Lake Prespa^3^ and Tenaghi Pilippon^42^) discussed in this paper. The map was generated using ArcGIS 10.2.2 (http://www.esri.com/software/arcgis).

Knowledge of the past climatic interaction of large-scale atmospheric systems controlling hydroclimate variability is of wide importance because it improves our understanding of global climate evolution and may help constrain predictions of future climate changes. Nevertheless, it is still challenging to reconstruct the dynamics and impact of past large-scale atmospheric circulation systems. This is especially true over the European continent because Europe receives competing influences of the Atlantic, Mediterranean and continental climates, and palaeoclimate proxies preserving the imprint of such climatic interaction signals are not intensively studied or more often, difficult to quantify. Probably the best region to study past climate conditions related to the coherence of those climatic regimes is Southeastern Europe (Fig. 1), where these climate influences are interacting^1–3^. Therefore, past changes in the strength and dynamics of such circulation modes are expected to be recorded in palaeoclimate archives from this region. Additionally, one of the oldest known fossils of Anatomically Modern Humans (AMH) in Europe were found in this area in the Peştera cu Oase cave (Western Romania; Fig. 1) and dated to ~40,000 years BP^4^. As such, this region may be regarded as a key area for understanding the relationship between past environmental changes in this area and the dispersal of AMH throughout Europe during the late Marine Isotope Stage (MIS) 3.

In Southeastern Europe, loess-palaeosol sequences are one of the most important and usually the only available terrestrial archives of Quaternary palaeoclimate dynamics^1, 2, 5–8^. Although loess from Southeastern Europe has been in the focus of recent research, information about interactions among large-scale atmospheric systems that played a significant role in loess formation are scarce^2^. In our study, we reconstructed climatic conditions in the Lower Danube region during the past ~50,000 years using high-resolution grain-size, environmental magnetic and geochemical data supplemented by luminescence dating. Grain-size distributions reflect changes in aeolian dynamics, sources of aeolian dust and pedogenesis^2, 9, 10^, whereas environmental magnetism indicates the post-depositional formation of ultrafine magnetic particles during *in-situ* weathering and pedogenesis, both linked to variations in soil humidity^11, 12^. Geochemical characteristics of the sediment can indicate the provenance or potential changes in the source area, and also give information on the weathering intensity. Based on the comparison between palaeoclimatic datasets from different regions in Europe we reconstruct the temporal and spatial interactions of Atlantic and continental climatic systems over Europe during the studied time period. Moreover, we evaluate the relations of inferred past atmospheric systems dynamics and their possible influence on the AMH dispersal throughout Europe.

## Regional setting and study sites

Geomorphologically, Southeastern Europe is a diverse region, mainly mountainous throughout the Balkan area (Dinarides, Rhodope, and Balkan Mountains), whereas to the North the Carpathians separate two large lowland basins, the Middle Danube (Carpathian) Basin in the West and the Lower Danube (Walachian) Basin in the East, coinciding also with the westernmost extent of the Eurasian steppe belt (Fig. 1). This area likely experienced major changes in the relative influence of large-scale atmospheric systems during the Middle and Late Pleistocene because of such geographical and geomorphological conditions^2, 5, 13^. It is suggested that during the last glacial cycle the outermost extent of the Mediterranean climate regime was mainly limited to the Balkan Peninsula^2^. Conversely, the Middle and Lower Danube Basins were mostly under the influence of Atlantic and continental climates. The continental climate is characterized by expressed seasonality and moderate precipitation (concentrated mostly in the warmer months), while the Atlantic climate influence is related to the Westerlies. Similar to present-day conditions, the Lower Danube Basin was permanently under stronger continental climatic conditions during the Middle and Late Quaternary, when compared to the Middle Danube Basin^5^. Hence, a more detailed understanding of differences in the palaeoclimate evolution of the Middle and Lower Danube Basins provide information on temporal dynamics and spatial interaction of continental and Atlantic climates. Loess sequences from the Middle Danube Basin were studied intensively over the past decades^1, 6, 14^. However, besides notable advances in luminescence dating^15, 16^, the Lower Danube Basin lacks high-resolution proxy data for the last glacial cycle. We performed high-resolution sediment analyses on the Urluia and Vlasca loess sections from the Lower Danube Basin, Romania (Fig. 1), for the past ~50,000 years. The sections were sampled in 2 cm increments for sedimentological, petrophysical and geochemical analyses. In this study, sediment fine fractions (<5 µm), the frequency dependent magnetic susceptibility (χ_fd_) and weathering indices derived from geochemical analyses are used as an indicator of increased pedogenesis and weathering intensity, while the U-ratio (coarse/fine silt (16–44/5.5–16 µm))^10^ is taken as an indicator of the wind strength.

## Results

**Figure 2:**
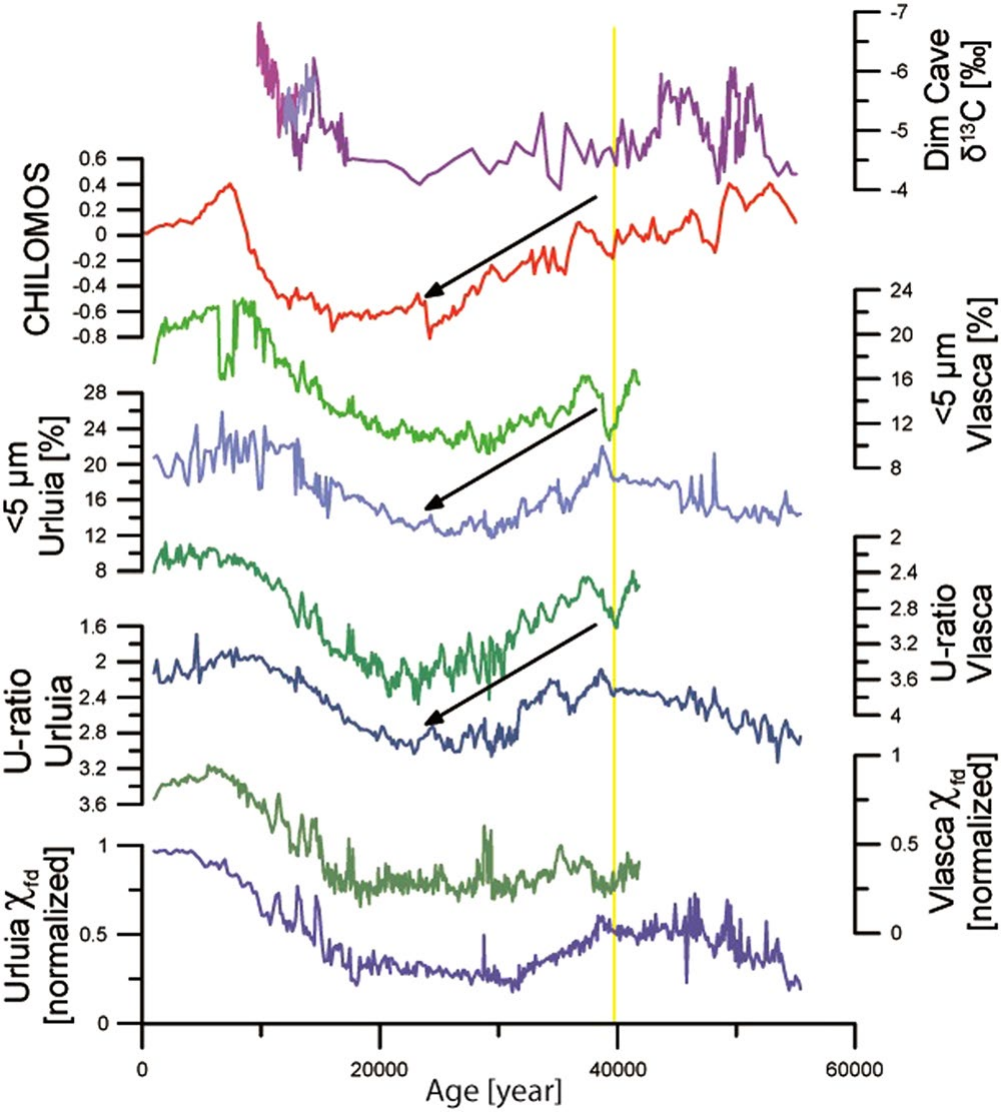
Direct comparison between proxies from the Urluia and Vlasca sections represented by U-ration, fine particles (<5 µm) and χ_fd_ (values are normalized), and their comparison with a stacked climatic record from northern China (CHILOMOS^34^) and δ^18^O record from Dim Cave^36^ over the past 55,000 years. The straight yellow line represents the timing of the Campanian Ignimbrite/Y-5 tephra deposition. Black arrows indicate a trend of general continentalization.

**Figure 3:**
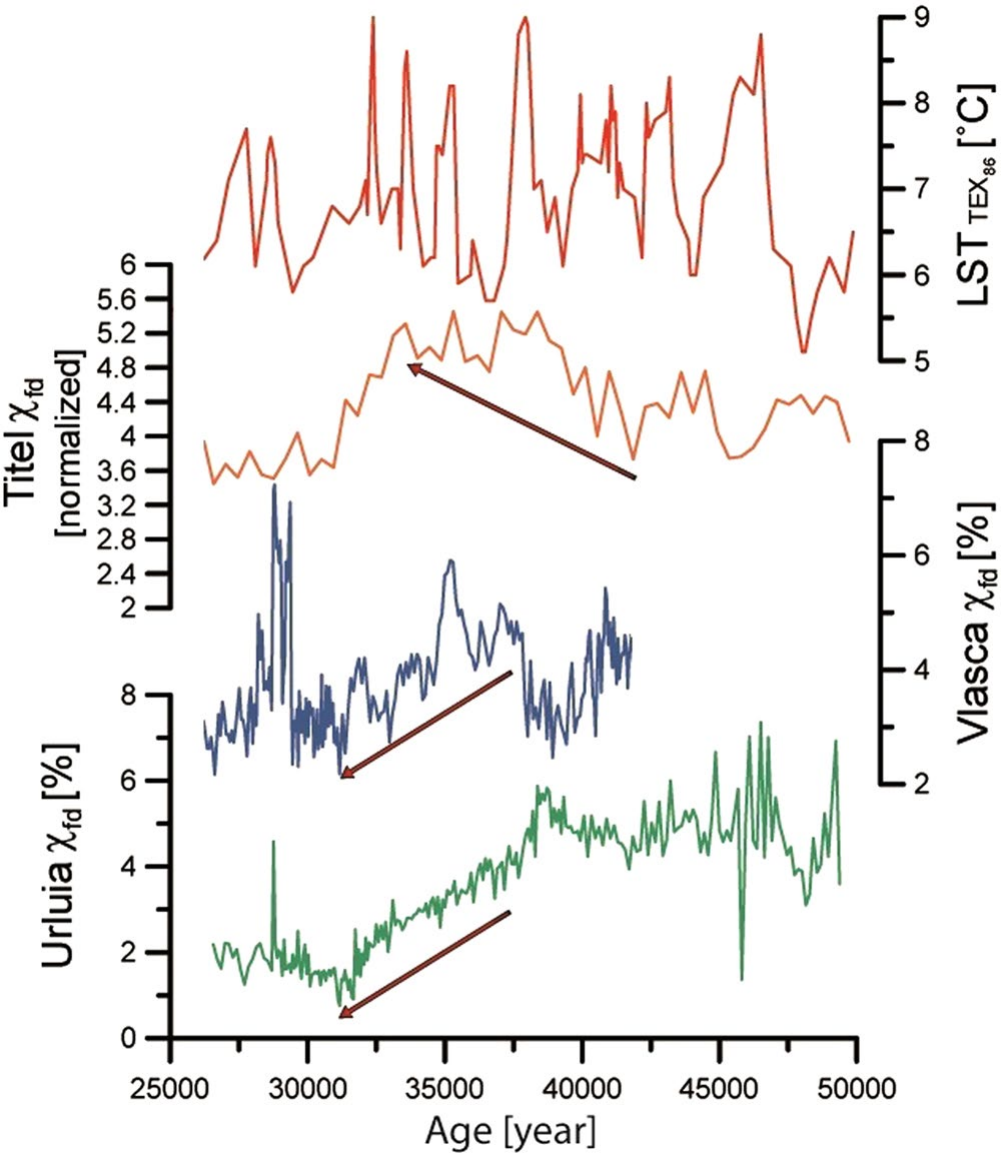
Direct comparison between χ_fd_ record from Urluia (green line) and Vlasca (blue line), χ_fd_ from Titel loess-palaeosol sequence^37^ (orange line; Middle Danube Basin) and their comparison with the mean annual lake surface temperature (LST; red line) of the Black Sea^26^ over 25,000–50,000 years ago.

The most relevant proxy data of the Urluia and Vlasca sections are presented in Figs 2 and 3, while the stratigraphy and more detailed descriptions are given in the Supplementary Information. Both sections are comprised of loess and are silt-dominated, with a particular domination of coarse silt. The Vlasca section is characterized by a higher contribution of coarse particles with the average mean grain-size being 44.5 µm (Fig. S5) and an average sand content of 22.0%. The Urluia section is composed of slightly finer particles (average mean grain-size 37.9 µm and average sand content of 17.9%; Fig. S4). The contribution of fine particles (<5 µm) varies from 9.3 to 23.2% (average 13.8%) at Vlasca and from 11.7 to 30.2% (average 16.1%) at Urluia (Fig. 2). The U-ratio is in the range from 2.1–3.9 at Vlasca and varies between 1.7 and 3.3 at Urluia (Fig. 2). The lowermost part of the Vlasca section (from the bottom to 10.1 m depth) is represented by a sandy layer (up to 58.3% sand) and it is probably related to Danube River activities, and therefore is excluded from the palaeoclimate reconstruction.

The mass specific magnetic susceptibility (χ) from the Vlasca section varies between 25.3 * 10^−8^ and 111.6 * 10^−8^ m^3^/kg (average 39.7 * 10^−8^ m^3^/kg) and for the Urluia section from 17.5 * 10^−8^ to 86.6 * 10^−8^ m^3^/kg (average 28.4 * 10^−8^ m^3^/kg; Fig. S6). The frequency dependent magnetic susceptibility (χ_fd_) at Vlasca varies in a wide range between 1.8 and 11.3% (average 4.7%) and at Urluia between 2.5 and 11.7% (average 4.2%; Figs S4–S6). It is important to note an offset in χ (Fig. S6) and χfd (Figs S4 and S6) at the Urluia section between the oriented samples that preserved their original moisture content and structure, and the sediment samples that have been dried, gently homogenized and compressed during the preparation process. Although all samples were density normalized, there is a difference between the density normalized susceptibilities of loess that preserved its original structure and moisture in oriented samples and such of dried and compressed loess. Although density normalized values are higher for non-oriented samples (Figs S4 and S6), the general trends between the records are the same. Therefore, the values are normalized to a common base when presenting the whole profile of Urluia (Fig. 2) to avoid issues in displaying data.

**Figure 4:**
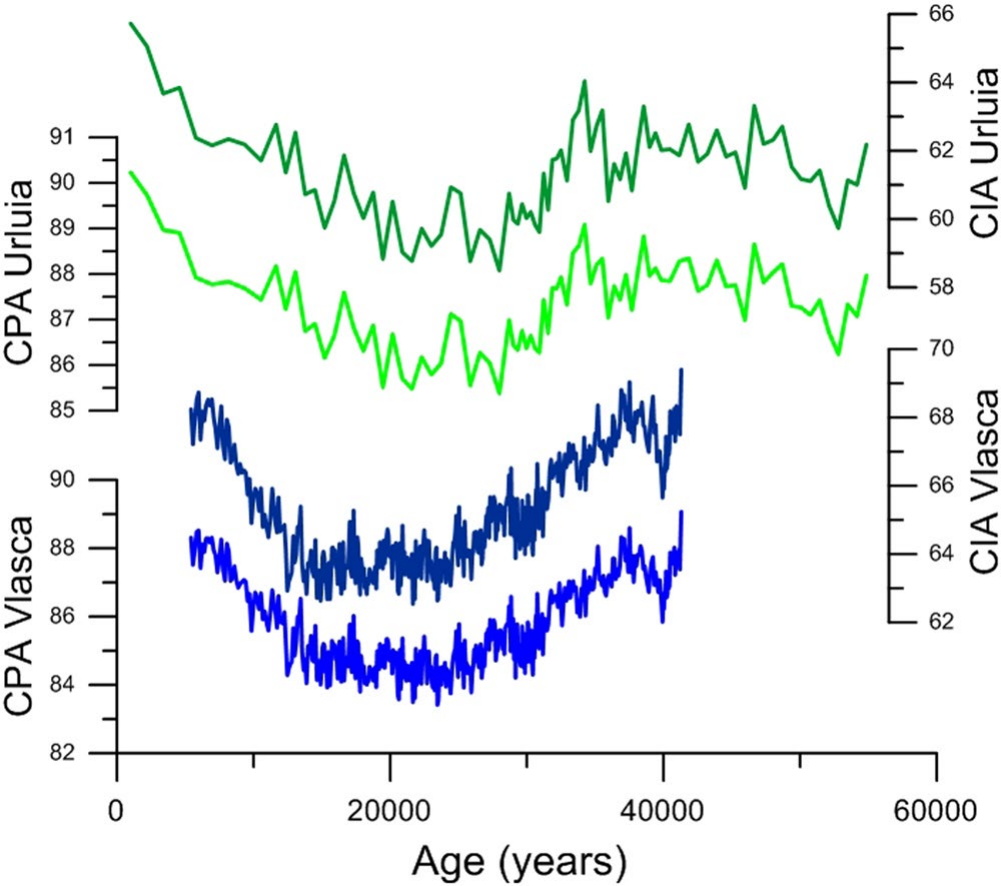
Weathering indices (CIA and CPA) for the Urluia and Vlasca sections.

Detailed geochemical investigations were performed at Urluia (every fifth sample was measured allowing the resolution of 10 cm) and Vlasca (with higher resolution of 2 cm increments, but with a focus on the samples between 41,500 and 5,500 years ago) sections. Both sections show a generally similar geochemical composition. The sediments are dominated by SiO_2_, oscillating between 50.7 and 66.2% (average 58.0%) at the Urluia section and between 50.7 and 66.2% (average 57.8%) at the Vlasca section. CaO (for Urluia between 4.8 and 24.4%, average 14.7%; for Vlasca between 9.2–19.8%, average 13.8%), Al_2_O_3_ (average for Urluia 13.5% and for Vlasca 13.3%), FeO (average for Urluia 5.0% and for Vlasca 4.7%), MgO (average for Urluia 4.0% and for Vlasca 4.6%), K_2_O (average for Urluia 2.3% and for Vlasca 2.2%), Na_2_O (average for Urluia 1.2% and for Vlasca 1.3%) and TiO_2_ (average for Urluia and Vlasca is 1.0%) are also major contributors to the geochemical composition of sediments (Fig. S9), while all other elements comprise less than 1%. Geochemical investigations are also often used for better understanding of weathering intensity^17–19^. Numerous ratios between soluble and mobile elements and immobile and non-soluble elements are widely used to give an insight into the weathering intensity of loess. Those ratios rely on the selective removal of soluble and mobile elements from a weathering profile compared to the relative enrichment of immobile and non-soluble elements^17, 18^. Among those, weathering indices such as the Chemical Index of Alteration^20^ (CIA = (Al_2_O_3_/(Al_2_O_3_ + Na2O + CaO* + K2O)) * 100; CaO* is silicatic CaO) and the Chemical Proxy of Alteration^18^ (CPA = (Al_2_O_3_/(Al_2_O_3_ + Na2O)) * 100) are commonly used and widely accepted as reliable weathering indices in most environments (Fig. 4). At the Urluia section, the CIA is in the range between 58.4 and 65.7 and CPA between 85.3 and 90.2, while at the Vlasca section CIA varies between 62.5 and 69.3 and CPA between 83.4 and 89.0 (Fig. 4). Fluctuations of CIA and CPA show similar behaviour at both sections (Fig. 4).

Scanning electron microscope images of the glass shards (and also other magmatic and detrital aeolian grains) from a tephra layer at the Vlasca section are presented in Fig. S3, while Table S2 shows the geochemical composition of tephra glass shards. According to the geochemistry of the glass shards, this layer is unambiguously related to the Campanian Ignimbrite/Y-5 tephra.

### Luminescence dating

Seven samples from the Urluia section were luminescence dated using the post infrared infrared protocol at 290 °C^21^. The samples show bright signals and all aliquots (10–24 per sample) passed the SAR rejection criteria. The prior IR stimulation temperature test of sample C-L3702 shows a plateau for temperatures between 50 °C and 170 °C (Fig. S1). Dose recovery tests (DRTs) are within 10% of unity for all samples (Fig. S1). Residual doses are <10 Gy and were subtracted for DRTs, but not for equivalent dose (D_e_) measurements. Fading measurements show variable results with a mean g2 days = −0.9 ± 0.7%. D_e_ distributions show low relative standard errors <6%. Moreover, overdispersion values calculated by the central age model (CAM)^22^ are small (<5%), only sample C-L3715 shows a higher value of 9.4 ± 1.6%. α-efficiency measurements determined a mean a-value of 0.136 ± 0.02. Ages and D_e_s increase with depth from 21 ± 1.6 ka (84.3 ± 4.3 Gy) to 54.2 ± 4.1 ka (258.2 ± 13.3 Gy). Dose rates range from 4.0 ± 0.2 Gy/ka to 5.3 ± 0.3 Gy/ka. A summary of all relevant luminescence data is given in Table S1 and Fig. S2.

## Discussion

The chronologies of both sections are primary based and linked to the occurrence of the Campanian Ignimbrite/Y-5 tephra dated to 39,930 ± 100 years BP^23^. This layer serves as an excellent chronological marker horizon for loess deposits in the Lower Danube area^23^. The age models used here are based on the ages obtained by luminescence dating and correlative techniques. Detailed information on establishing the age models are presented in the Supplementary Information.

Geochemical compositions of the Vlasca and Urluia sections are similar to other Danubean loess-palaeosol sequences^7, 24, 25^, and the geochemical results from both sections do not indicate any major change in provenance and source area, especially between 50,000 and 30,000 years ago (Fig. S10). The grain-size distributions from both sections are characterized by a higher contribution of coarser particles for loess sediment (Fig. 2, S4 and S5). The Vlasca section is situated nowadays in the immediate vicinity of the Danube River (Fig. 1) and it was likely also during the past 50,000 years under a strong influence of short-distance transported material, which is supported by the coarse particle contribution (Fig. S5). The Urluia section is more distant from the Danube (Fig. 1), and was subjected to receiving more distant material than the Vlasca section, although generally coarse particles still indicate a proximity to a source area (Fig. S4). Similar patterns in grain-size distribution between Vlasca and Urluia indicate that the recorded signal reflects a consistent regional pattern (Fig. 2). The grain-size distribution from both sections shows a higher contribution of fine particles before the Campanian Ignimbrite/Y-5 tephra deposition, indicating moderate wind dynamics from ~50,000–40,000 years ago. Upon the Campanian Ignimbrite tephra deposition, an increase in particles size and U-ratio values are recorded. Such a grain-size distribution indicates a clear trend towards stronger wind intensity and drier and probably colder climatic conditions during late MIS 3 (40,000–27,000 years ago) (Fig. 2). Low fine fraction contribution and high U-ratio values during MIS 2 indicate pronounced arid and cold conditions (Fig. 2).

Overall, both sections show changes in soil humidity indicated by variations in χ_fd_ that reflect a pacing similar to Dansgaard-Oeschger millennial-scale past climate variability (Fig. S6). However, due to the limitations in dating techniques in loess research, it is still not possible to reliably match fluctuations in loess records to such short climatic events. A comprehensive discussion on this issue is presented in Supplementary Information. Moreover, the χ_fd_ record from Vlasca seems to reflect changes in humidity with higher amplitude than Urluia (Figs 2 and 3). This may be due to the local influence of the Danube River at Vlasca, whereas Urluia reflects the more regional humidity pattern. Although χ_fd_ shows differences in the amplitudes, the general trends and patterns are comparable and are in good agreement with the nearby Rasova section^12^ (Fig. S7). Records of χ_fd_ from both sections indicate that the period between 50,000 and 40,000 years ago was characterized by enhanced moisture. However, shortly after the Campanian Ignimbrite/Y-5 tephra deposition, χ_fd_ records suggest a trend of decreasing humidity over late MIS 3 in the Lower Danube Basin, also observed in fine grain-size fractions (Figs 2 and 3). Trends of decreasing humidity and consequently weaker weathering are additionally supported by the decreasing trends of CIA and CPA during the late MIS 3 (Fig. 4). The mean annual lake surface temperature of the Black Sea^26^ does not show any similar trend of decreased humidity and/or temperatures during late MIS 3 (Fig. 3). This suggests that a change in trend towards climatic deterioration and progressive continentalization of the Lower Danube Basin is related to a change in atmospheric circulation, rather than in changes in the Black Sea mean annual lake surface temperature. However, the long-term glacial vegetation dynamics inferred from a pollen record from the Black Sea sediment core specify the period from ~40,000–32,000 years BP as a major arid phase^27^. Thus, the general continentalization trend during late MIS 3 is clearly observed in the terrestrial environments surrounding the Black Sea.

During MIS 2, the Lower Danube Basin experienced dry and cold environmental and climatic conditions as indicated by a low fine fractions contribution, decreased χ_fd_ values (Fig. 2) and low weathering as indicated by CIA and CPA (Fig. 4). However, late MIS 2 shows a sharp increase in χ_fd_ reaching the highest values in the Holocene, indicating a noticeable increase in regional humidity with the onset of the deglaciation (Fig. 2). Accordingly, the Lower Danube Basin experienced relatively mild conditions during middle MIS 3 (50,000–40,000 years ago), while late MIS 3 was exposed to the general deterioration of the environmental and climatic conditions, with pulses of increased humidity possibly related to interstadial phases. Early MIS 2 and the LGM were generally dry and cold with increased wind intensity, while the deglaciation period is characterized by a sharp increase in humidity and a weakening of wind strength.

Our data indicate that the onset of a general continentalization (less precipitation and colder winters) of climate in the Lower Danube Basin became a persistent feature from ~40,000–27,000 years (Figs 2–4). Nevertheless, the observed continentalization is not only a regional feature of the Lower Danube Basin. The ELSA (Eifel Laminated Sediment Archive) Vegetation-Stack^28^ from Western Europe also indicates a trend of continentalization, culminating as a shift from boreal forest to steppe conditions at ~36,500 years ago (Fig. S11). This implies that large areas of Eastern and Western Europe experienced a major environmental change towards open steppe within late MIS 3. The continentalization of major parts of Europe (from east to west) can be explained by the increase in the Fennoscandian ice sheets or with the general increase of the Eurasian high-pressure system. Recent studies report the Fennoscandian ice sheet to a very limited extent before ~35,000 years ago, while the major expansion started only after ~30,000 years ago^29, 30^. Moreover, some studies even argue for an ice-free MIS 3 Fennoscandia^31^. Accordingly, an increase in the extent of the Fennoscandian ice sheet was not a forcing mechanism for the observed changes in atmospheric circulation.

It is widely accepted that increases in grain-size seen in records from the Chinese Loess Plateau are linked to a strengthening of the East Asian winter monsoon due to an intensification of the Eurasian high pressure system (the Siberian High)^32, 33^. Therefore, the grain-size record from the Chinese loess sequences is a reliable indicator of the Siberian High intensity. The Chinese loess records show a clear trend of increase in grain-size during late MIS 3^33–35^ (Fig. 2), indicating a strong increase in the Siberian High intensity during this time period. The similarities in the grain-size trends between the Lower Danube loess sections discussed in this study and data from the Chinese Loess Plateau over late MIS 3 (Fig. 2) support that a common Eurasian atmospheric forcing pattern was responsible for the climatic evolution of these two regions during that time period. Therefore, we argue for an increased influence of the Eurasian high-pressure system (the Siberian High) as the determining factor for palaeoclimate over major parts of Eurasia, including the Lower Danube Basin, during late MIS 3. This for the first time suggests that the Siberian High had a crucial influence on European climate regimes during the time period when the Fennoscandian ice sheets still had a limited extent^29, 30^.

The increasing influence of the Siberian High on Europe must have had a strong influence on prevailing air masses from the Atlantic (Westerlies) during the limited extent of the Fennoscandian ice sheet. The speleothem record from the Dim Cave^36^ (Fig. 2) suggests a shift of the Westerlies from the European track (NW-SE European trajectory across the Balkans) towards the Mediterranean track (W-E trajectory where the air mass passes over the Mediterranean Sea) during late MIS 3 (Fig. 2). This shift in the course of the Westerlies can be explained with an increased influence of the Siberian High on Europe. Increasing air pressure over Eastern and Western Europe during late MIS 3 caused a shift of the Westerlies with lower air pressure to the south.

**Figure 5:**
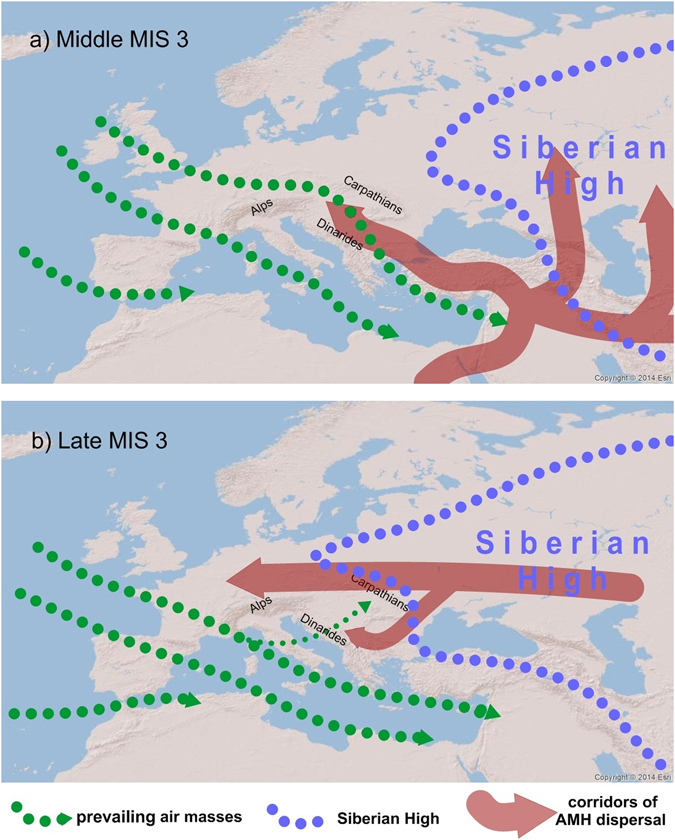
(**a**) Simplified scheme of proposed general atmospheric circulation patterns over Europe during middle MIS 3. Note that the Westerlies were reaching Central and Southeastern Europe via the NW-SE trajectory; (b) Simplified scheme of proposed general atmospheric circulation patterns over Europe during late MIS 3. Increase Siberian High influence on Europe had a major influence on Eastern and Western Europe, while the Westerlies shifted to the S-E trajectory, bringing the warmer air masses from the Mediterranean to the Balkans and the Middle Danube Basin. The blue line represents a schematic intensification of Siberian High, green lines show prevailing air masses and red lines represent paths of AMH dispersal in Europe. The map was generated using ArcGIS 10.2.2 (http://www.esri.com/software/arcgis).

Contrary to observations of general continentalization from the Lower Danube Basin, loess records in the Middle Danube Basin indicate an opposite trend (Fig. 3)^1, 37–40^. The Middle Danube Basin is characterized by an increase in finer particles and in χ_fd_ after ~40,000 years ago^1, 37, 39, 40^, pointing to warmer and more humid conditions during late MIS 3 (Fig. 3). Such an opposing climatic evolution between these areas support the interpretation of a southwards shift of the Westerlies after ~40,000 years ago^36^. The prevailing wind track that was dominant before ~40,000 years had a NW-SE trajectory^36^ bringing the colder air masses from the north over the Middle Danube Basin (Fig. 5a). A shift towards the Mediterranean track induced warmer and moist air masses that were present in the Mediterranean^41, 42^ and the Balkans^2^ to reach the Middle Danube Basin in late MIS 3 (Fig. 5b). Warm and moist air masses did not reach the Lower Danube Basin due to the orographic obstacle of the mountain chains throughout the Balkans and particularly the Carpathian Mountains (Figs 1 and 5b). Summarizing, we highlight that during the limited extent of the Fennoscandian ice sheets the Siberian High played a crucial role on the evolution of prevailing atmospheric circulations and palaeoenvironmental conditions over Europe.

Such atmospheric circulation over Europe may have had an important role for AMH dispersal into Europe. The first AMH appeared in Europe roughly 45,000 to 40,000 years ago^43, 44^, and the reasons for the timing of the AMH dispersal have widely been debated. While some studies highlight the continuous presence of native Neanderthals before their demise at around 40,000 years ago (potentially due to the impact of Heinrich event 4 and the Campi Flegrei super-eruption) as an important obstacle for earlier AMH dispersal^45, 46^, others report AMH cultural layers to be present in Europe before that time period^47–49^. However, none of these studies advanced a robust explanation for the AMH dispersal and observed replacement of other hominid species. Although it is likely that the AMH reached Europe before ~40,000 years ago, the major dispersal of our ancestors and the final demise of the Neanderthals from Europe occurred shortly after ~40,000 years ago^50^ (although it is suggested that Neanderthals survived in some parts of southern Iberia^51^ and the Balkans^52^ until ~32,000 years BP).

Based on the similarities of techno-complexes from lithic assemblages, a commonly proposed corridor of AMH dispersal into Europe is over the Levant and Balkans^49, 53^. However, AMH have been demonstrated as already present in western Siberia^54^ and on the Don River^55^ in present-day Russia roughly around 45,000 years ago. These finds suggest that the AMH were present in Siberia well before the major dispersal into Europe took place. This highlights the possibility of a northern corridor, over the eastern and northern parts of the Black Sea and the Eastern European Plains. According to techno-complexes, this corridor was likely not the main path of AMH dispersal before ~40,000 years. However, the Campi Flegrei super-eruption and the deposition of Campanian Ignimbrite/Y-5 ash at around 39,930 years ago had a strong impact in this region when compared to the Levant, since thick tephra deposits are reported from the Mediterranean to the Eastern European Plains^46^. The deposition of several centimeters of volcanic ash as modelled by Marti *et al*.^46^ or the field evidence of tens of centimeters thick ash beds along the Danube and its side valleys^23, 56^ must have had a devastating impact on flora and fauna in this region. This may permanently annul or decrease the advantage of primary occupation that Neanderthals had over Southeastern and Eastern European Plains. Based on simulations to define eco-cultural niches associated with Neanderthal and AMH adaptive systems during alternating cold and mild phases of MIS 3, it is suggested that during the following Greenland Interstadial 8 (around 38,000 years ago), AMH expansion resulted in competition with which the Neanderthal adaptive system was unable to cope^50^. Accordingly, since the Campanian Ignimbrite/Y-5 tephra deposition annulled the advantage of primary occupation, AMH had an advantage in reoccupying the areas affected by the tephra deposition. With an increase in the Siberian High intensity after the Campi Flegrei super-eruption, as reported in this study, intensive westwards dispersal of AMH from western Siberia may have taken place over the Eastern European Plains (Fig. 5). Here we suggest that although the first AMH may have used the southern corridor over Balkans to enter Europe, during late MIS 3 (in particular after the Campanian Ignimbrite/Y-5 eruption) the corridor over the Eastern European Plains was likely the most dominant one for the AMH dispersal into Europe. The genetic study of the first known European AMH from Peştera cu Oase cave shows that this population did not contribute substantially to later humans in Europe^57, 58^, supporting a later arrival of AMH with different origin via the northern corridor. The permanent intensification of the Siberian High during late MIS 3 caused a dryer and colder environment (especially cold winters) that became unfavourable for the survival of AMH in western Siberia. With the temporal intensification of the Siberian High adverse environmental and climatic conditions prevailed in Eastern European Plains, but the palaeoenvironment in major parts of Europe became an open and fertile steppe (Fig. S11) able to sustain large herds of herbivores and their hunters. Such climatic and environmental changes initiated a significant dispersal of AMH from western Siberia and Eurasian interior towards Western Europe.

## Material and Methods

To obtain the grain-size data, subsamples of 0.1–0.3 g fine-earth (<2 mm in size) were pre-treated with 0.70 ml of 30% hydrogen peroxide (H_2_O_2_) at 70 °C for 12 hours. This process was repeated until a bleaching of the sediment occurred, but not longer than three days. To keep particles dispersed, the samples were treated with 1.25 ml, 0.1 M sodiumpyrophosphate (Na_4_P_2_O_7_ * 10H_2_O) for 12 h^2, 7^. Particle size characteristics were measured with a LS 13320 Laser Diffraction Particle Size Analyser (Beckman Coulter). To calculate the grain-size distribution the Mie theory was used (Fluid RI: 1.33; Sample RI: 1.55; Imaginary RI: 0.1)^59–61^.

Bulk samples for environmental magnetism were dried and packed into plastic boxes, and subsequently compressed and fixed with cotton wool to prevent movement of sediment particles during measurement. For Urluia, the majority of samples (323) were collected as oriented samples, using brass tubes and an orientation holder. This way, samples were placed in the diamagnetic boxes directly in the field. The volumetric magnetic susceptibility was measured at frequencies of 300 and 3000 Hz in a static field of 300 mA/m using a Magnon International VSFM. Data were corrected for drift and for the effect of sampling boxes (weak diamagnetism) and normalized to density. Hence, magnetic susceptibility is given as mass specific susceptibility in m^3^/kg. The frequency dependence was calculated as χ_fd_ = (χ_lf_ – χ_hf_)/χ_lf_  * 100 [%]^2, 11, 12^.

For geochemical analyses, all bulk sediment samples were sieved down to 63 μm and dried at 105 °C for 12 hours. An 8 g quantity of the sieved material was mixed with 2 g Fluxana Cereox wax, homogenized and pressed to a pellet with a pressure of 19.2 MPa for 120 seconds. The measurements were conducted by means of a pre-calibrated method. Samples were analysed for major and trace element abundances with polarization energy dispersive X-ray fluorescence (EDPXRF) using a SpectroXepos.

For investigating glass shard major oxide chemical composition, the Campanian Ignimbrite/Y-5 tephra sample (from the visible tephra layer) was sieved and the residual was mounted in epoxy resin, ground and polished. Measurements were made using single-grain, wavelength-dispersive electron microprobe analysis at the Bayerisches GeoInstitut on a Jeol JXA8200 microprobe employing an accelerating voltage of 15 keV, a 6 nA beam current and defocused beam. Order of measured elements (first to last): Na, Si, K, Ca, Fe, Mg, Al, P, Ti, Mn, Cl, with peak counting times averaging 10 s for Na, 30 s for Si, Al, K, Ca, Fe and Mg, 40 s for Ti and Mn, and 60 s for P. Precision is estimated at <1–6% (2σ) and 10–25% (2σ) for major and minor element concentrations, respectively.

Luminescence dating was performed on seven samples from the Urluia section. For equivalent dose (D_e_) determination, fine-grained (4–11 μm) polymineral samples were measured in a Risø TL/OSL DA 20 reader at the Cologne Luminescence Lab. The post infrared infrared stimulated luminescence (pIRIR) protocol by Thiel *et al*.^21^ and the central age model^22^ were used. Prior IR stimulation temperature tests, dose recovery tests, residual and fading measurements were conducted. Radionuclide concentrations were measured in a high-purity germanium gamma-ray spectrometer and converted into dose rates. Additional details on the methodology are presented within the Supplementary Information.

## Electronic supplementary material

Supplementary Information
