# Supplementary material for: Shift of large-scale atmospheric systems over Europe during late MIS 3 and implications for Modern Human dispersal
Source: Sci Rep. 2017 Jul 19;7:5848. doi: 10.1038/s41598-017-06285-x (PMC5517514; doi:10.1038/s41598-017-06285-x)
Supplement: Supplementary file 1 — Supplementary Information [file 41598_2017_6285_MOESM1_ESM.pdf]

Supplementary Information

Shift of large-scale atmospheric systems over Europe during late MIS 3 and implications for Modern Human dispersal

Igor Obreht, Ulrich Hambach, Daniel Veres, Christian Zeeden, Janina Bösken, Thomas Stevens, Slobodan B. Marković, Nicole Klasen, Dominik Brill, Christoph Burow, Frank Lehmkuhl

1. Sampling strategy

The samples from the Urluia section were collected during a field campaign in spring 2014; the Vlasca section was sampled in autumn 2015. Profiles were freshly cleaned to allow for continuous, high-resolution, incremental sampling. Samples for sedimentology and environmental magnetism were taken in 2 cm increments. At the Urluia section, samples from 2.04 m to 8.50 m depth were taken as oriented samples for future palaeomagnetic studies. Oriented samples were not taken in the upper 2.04 m because strong bioturbation occurs ranging from mm to dm scale. Generally, during sampling large scale bioturbation structures (e.g. krotovinas) were avoided. Moreover, seven luminescence samples were taken at Urluia by hammering and extracting 15 cm long metal tubes into the sediment wall, and by collecting the surrounding material in a radius of 30 cm for dose rate determination.

2. Stratigraphy of studied profiles

2.1.Urluia

The Urluia loess section (44.09417°N, 27.9031°E, 125 m a.s.l) is located on the southern Dobrogea plateau in southeastern Romania. In this area, up to few tens of m thick loess-palaeosol sequence covers Cretaceous limestone uplifted in Early to Mid Quaternary[1]. The outcrop face at Urluia is ca. 800 m long and strikes NNW-SSE. This study focuses on the upper part of the loess sequence exposed in an abandoned quarry.

The top of the section is formed by a well-developed ~1 m thick dark greyish-brown steppe soil followed by a diffuse transition towards loess from ~1.00-1.20 m, characterized by a transition towards lighter sediment and a decline of crumbling soil structure. From 1.20-1.70 m, yellow to yellow-brownish loess with a homogenous structure is exposed. This layer is rich in pseudo-mycelia (small sub-mm/dm-scale) carbonate precipitation following mm-sized biogalleries) and krotovinas (cm to sub-dm sized burrows of steppe mammals) whose frequencies decreases with depth. Several loess dolls (pedogenetic carbonate concretions) are observed between 1.45-1.65 m, albeit they are notably present until 2.50 m. From 1.70-2.40 m, the loess becomes less brownish and more yellowish with depth, while below 2.40 m it becomes yellowish. The upper 2.50 m of the profile are generally strongly bioturbated on a cm to sub-dm scale. At ~3.55 m, a clear change in color is observed from yellowish to brown-yellowish. From 3.50-3.70 m loess is notably coarser in gain-size and darker than above. The loess below is generally homogenous until 5.15 m, and below this layer until 5.45 m loess is again brownish and the amount of root channels with humic in fillings increases. A diffuse ochre-colored horizon occurs between 5.45-5.65 m, followed bygreyish-yellow loess with brownish mm-sized spots spanning the section until 7.40 m depth. From 5.70-5.95 m a dm-scale horizontal krotovina filled with grey loess and surface soil material (not sampled) crosses the sampled wall. Along the loess wall at Urluia, in some parts the tephra layer is preserved in up to one meter thickness on palaeoslopes and a palaeodepression, and it can be

followed along most of the wall. This tephra is related to the Campanian Ignimbrite tephra layer[1,2]. The sampled profile was chosen to be at a position which is least influenced by slope processes. Therefore, on the sampled wall the tephra is not very clearly standing out. However, based on detailed lithological characteristics and inter-profile correlation along the outcrop walls, at a depth between 6.15-6.2 m a crypto-tephra layer is suspected. From 7.40-7.50 m loess is pale reddish-brown, while at ~8.10 m a diffuse and bioturbated but clear border towards grey underlying loess occurs.

2.2.Vlasca

The Vlasca section (44.39951° N, 27.86409° E, 37 m a.s.l.) is located in the Lower Danube Basin (Southeastern Romania), and was sampled in a loess cliff at the Danube between the villages Vlasca and Stelnica. The cliffs are 1.8 km long and are striking from SW to NE. The studied profile is in total 10.72 m high.

Also at Vlasca, the top of the section is formed by a well-developed 0.8 m thick generally dark greyish-brown steppe soil; a 0.09 m rather thin middle brown horizon at the top is followed by a crumbled to subpolyedric textured middle brown horizon until ~0.30 m. Sediment from ~0.30 m until ~0.50 m has the same features as above, but it is lighter in color (light grey brown). From 0.50-0.68 m, the crumbled structure decreases. Down to 0.68 m depth the sediment is strongly rooted. Below 0.68 m and until 0.80 m the rooting intensity decreases and a gradual change in color towards more brownish shades is observed. From 0.80-1.00 m, a fine powdery and lighter greyish-whitish loess layer is followed by a 0.12 m horizon with initial forming of loess dolls and decreasing frequency of whitish carbonate spots complete the base of the modern soil formation. From 1.12-1.39 m depth downward alight brown greyish powdery loess with subpolyedric crumbly matrix occurs. Ocher (to ocher-greyish in the lower part) typical loess follows below until 2.43 m depth, with abundant small krotovinas and a bigger one (ca. 10 cm in diameter) at ~1.60 m. From 2.43-3.40 m typical homogenous loess is exposed. This interval is followed by loess with a gentle increase

in more crumbled structure until 7.70 m. From 7.70 m to 9.00 m the sediment is dark-ocher to brown in color and mm-sized dark organic spots are observed. The upper boundary to a tephra layer is very diffuse, but the pure tephra layer is exposed from 9.00-9.05 m. Below the tephra, the loess is generally sandier, and from 9.05-9.25 m it has a yellowish-brown-grey color, while it becomes greyish and more cemented below. Brownish loess appears from 9.80 to 9.90 m. From 9.90 until the end of the profile loess is again greyish. From 10.10 m downwards, the sand content and the degree of cementation rapidly increases.

3. Luminescence methodology

3.1. Equivalent dose (D_e) measurements

Samples for De determination were prepared under subdued red illumination. When opening the steel cylinders, the first ~2 cm of sediment from both ends were removed. After drying the samples for 24 h at 50°C the gravimetric water content was calculated after weighing the samples and hence the loss of water. The dried samples were then treated with 10% hydrochloric acid, 10% hydrogen peroxide and 0.01 N sodium oxalate to remove carbonates, organic matter, and to disperse sediment aggregates. The 4-11 µm grain size fraction was isolated using Stokes' law and by removing the clay fraction (< 4 µm) via centrifuging.

Continuous wave optically stimulated luminescence measurements (CW-OSL) were carried out on a Risø TL/OSL DA 20 reader. The reader is equipped with a $^{90}Sr/^{90}Y$ β-source and infrared (IR) LEDs emitting at 870 nm (FWHM = 40 nm). A 410 nm interference filter was used for signal detection. The $pIR_{50}IR_{290}$ SAR protocol was used[3,4]. The signal was integrated using the first 2.4 s of the stimulation curve minus a background derived from the last 25.6 s. A prior IR stimulation temperature test[3] was performed on sample C-L3713. Furthermore, several dose recovery tests (DRTs) with the favorable prior IR stimulation temperature were performed using bleached samples (24 h solar simulator). Finally, a mean equivalent dose was

determined using the central age model (CAM) and the common age model (COM)[5]. Residual doses were assessed after bleaching the aliquots for 24 hours in a Hönle Sol2 solar simulator. Fading tests were conducted on sample C-L3711. For these, a laboratory beta dose of 186 Gy was used and the normalized luminescence signals were measured after different storage times between 100 and 800 minutes.

3.2. Dose rate measurements

Samples for dose rate determination were oven dried at 50 °C for at least 48 h, homogenized and packed into plastic cylinders for radionuclide measurements. Radionuclide concentrations were measured on a high-purity germanium gamma-ray spectrometer after a resting period of four weeks to compensate for radon emanation during pretreatment. The dose rates and ages were calculated using DRAC, v.1.2[6]. Conversion factors of Liritzis et al.[7] and an estimated water content of 10±5% were included. The internal β dose rate contribution was calculated by assuming a K content of 12.5±0.5%[8] and a Rb content of 400±100 ppm[9]. Attenuation factors of Bell[10] and Guerin et al.[11] were used. The cosmic dose rate was calculated following Prescott and Hutton[12] considering the geographical position, altitude, sample depth and density of the overlying sediments. The α-efficiency was determined for six aliquots of two samples. Aliquots were thermally annealed (480°C) before administering α-doses up to 200 Gy in a Freiberg Instruments Lexsyg Research device. The laboratory dose of the samples was determined using β-irradiation in a Risø reader (pIRIR protocol). Finally, the α-efficiency was calculated as the ratio of the measured β-dose divided by the given α-dose[13]. A linear function was used for fitting. A mean a-value was used for all samples.

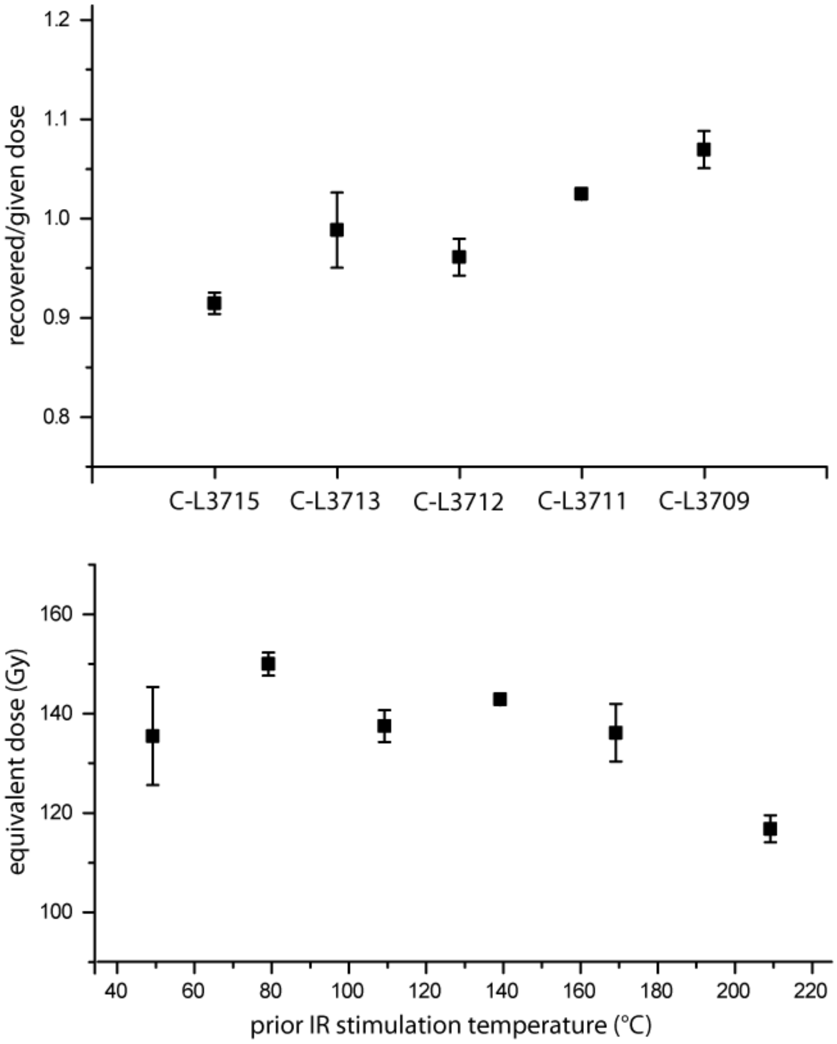

Figure S1. Dose recovery test (top) and prior IR stimulation temperature test (bottom). All tested samples are within the desired recovered/given dose ratio of 1.0±0.1. A plateau for prior IR stimulation temperatures is present between 50 and 170°C.

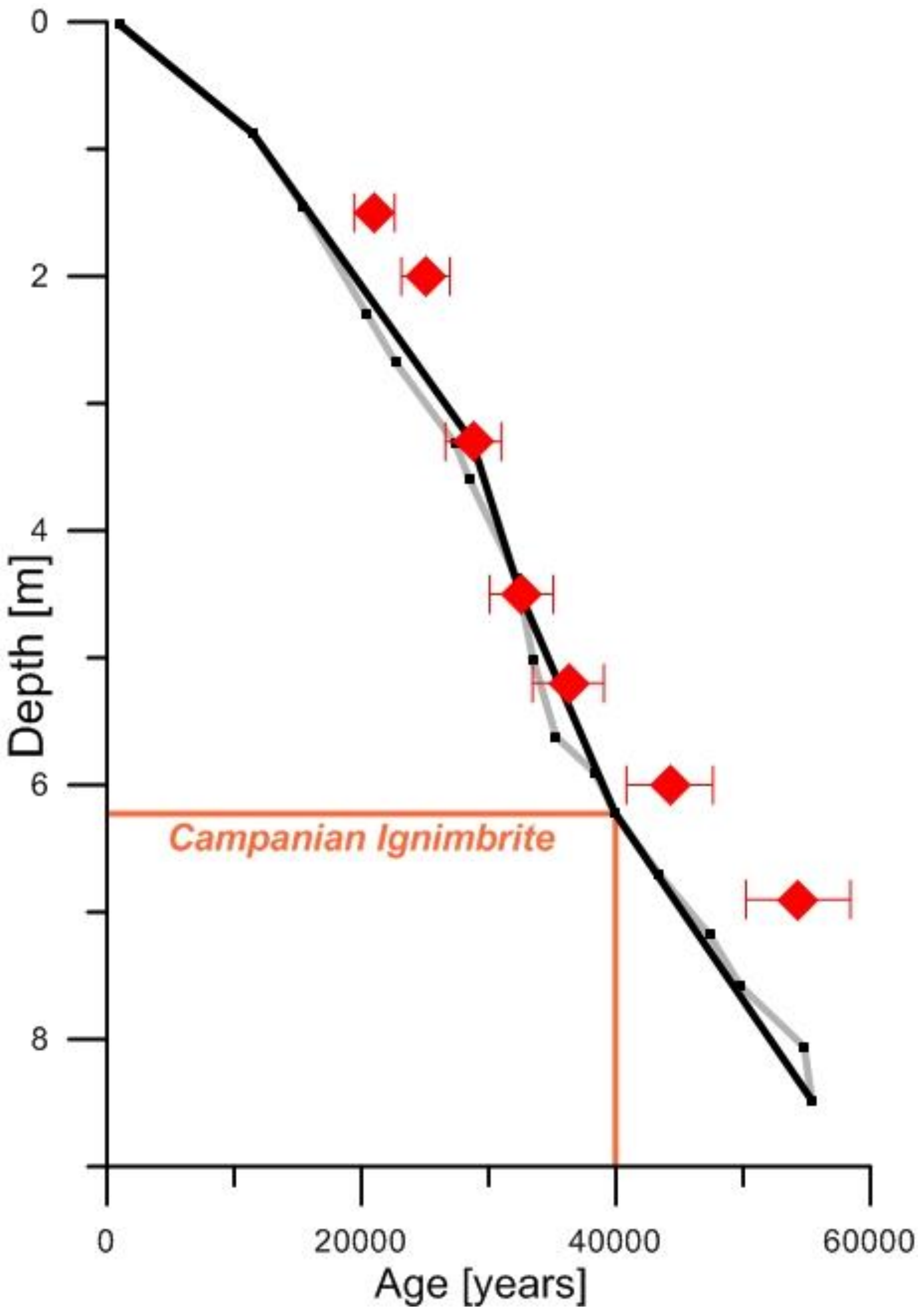

Figure S2. Consistency between luminescence ages (red squares and 1-σ uncertainty), correlative age model (grey line; black dots represent tie points) and the final age model (black line) of the Urluia section. Orange lines represent the timing and the depth of the Campanian Ignimbrite tephra layer.

Table S1. Summary of luminescence data for samples from Urluia (URL1). IRSL measurements were done on polymineralic fine grains (4-11 µm) on 9.8 mm aliquots using the $pIRIR_{290}$ protocol[3,4]. An internal K content of 12.5 ± 0.5 %[8] and Rb content of 400 ± 100 ppm[9] was assumed. The cosmic dose rates were calculated according to Prescott & Hutton[12], taking into consideration the altitude (125 m), latitude (44.094167°) and longitude (27.9031°) of the sampling site, as well as the density (1.7 g cm^{-3}) and thickness of the overlying sediments. Ages are presented with 1-σ uncertainty.

**Sample**	**Depth (m)**	n^b	**U (ppm)**	**Th (ppm)**	**K (%)**	$W_{measured}$ **(wt. %)**[c]	W_{used} **(wt. %)**[c]	$\dot{D}_{cosmic}$ **(Gy ka^{-1})**	$\dot{D}_{total}$[a] **(Gy ka^{-1})**	**DRT[d]ratio**	**Residual dose (Gy)**	D_e **(Gy)**	**OD (%)**[e]	**Age (ka)**
C-L3716	1.50	10/10	2.4±0.13	8.83±0.54	1.3±0.01	-	10±5	0.18±0.02	4.02±0.22	-	-	84.27±4.29	0.0±0.0	21.0±1.6
C-L3715	2.00	24/24	2.86±0.14	9.41±0.49	1.45±0.03	14.4	10±5	0.14±0.01	4.48±0.24	0.91±0.01	7.1±0.28	111.93±6.05	9.36±1.55	25.0±1.9
C-L3713	3.30	10/10	3.0±0.14	11.24±0.52	1.54±0.03	11.0	10±5	0.12±0.01	4.73±0.26	0.99±0.04	6.98±0.1	135.9±7.01	0.59±6	28.7±2.2
C-L3712	4.50	10/10	3.22±0.15	11.35±0.57	1.67±0.03	8.3	10±5	0.12±0.01	5.11±0.28	0.96±0.02	6.36±0.44	166.27±8.72	2.52±2.21	32.5±2.5
C-L3711	5.20	20/20	3.43±0.16	11.75±0.59	1.64±0.03	9.0	10±5	0.11±0.01	5.26±0.29	1.02±0.00	6.64±0.29	190.25±9.84	4.15±1.32	36.2±2.8
C-L3709	6.00	10/10	3.24±0.15	10.94±0.55	1.62±0.03	9.6	10±5	0.11±0.01	5.01±0.28	1.07±0.02	6.18±0.37	221.01±11.67	3.1±2.07	44.1±3.4
C-L3708	6.90	11/11	3.0±0.14	10.85±0.54	1.51±0.03	-	10±5	0.1±0.01	4.76±0.26	-	-	258.24±13.3	1.05±3.41	54.2±4.1

[a] a-value measured: 0.136±0.02

[b] Number of accepted in relation to measured aliquots.

[c] Gravimetric water content.

[d] DRT = Dose Recovery Test.

[e] OD = overdisperion, calculated using the Central Age Model[5].

4. Chronology and age model

4.1. Tephra chronology

At both sections a tephra layer was observed. At Urluia, this layer is only preserved as the crypto-tephra in the sampled main profile, albeit it can be clearly observed and followed over a major part of section wall using lithostratigraphic markers. Overall, the tephra layer is thicker in depressions and on slopes (up to one meter in some parts of the Urluia section[2]) of the primary relief at the time of deposition. Based on microprobe analyses and luminescence dating, previous investigations have related this layer to the Campanian Ignimbrite/Y-5 tephra[1,2,14].

Microprobe analyses of the tephra layer at Vlasca were performed on samples that were taken ~700 m northeast along the Danube bank from the studied section. These investigations were performed before the sedimentological sampling of the Vlasca section discussed here, in order to establish a chronology of the Vlasca loess site. Figure S3 shows the scanning electron microscope images of the glass shards, other magmatic and detrital aeolian grains, and Table S2 shows the geochemical composition of tephra glass shards. According to the geochemistry of the glass shards, this layer is unambiguously related to the Campanian Ignimbrite tephra. Consequently, chronologies of both sections are grounded on the Campanian Ignimbrite/Y-5 tephra, a reliable chronological layer dated to 39.93 ± 0.1 ka BP[15].

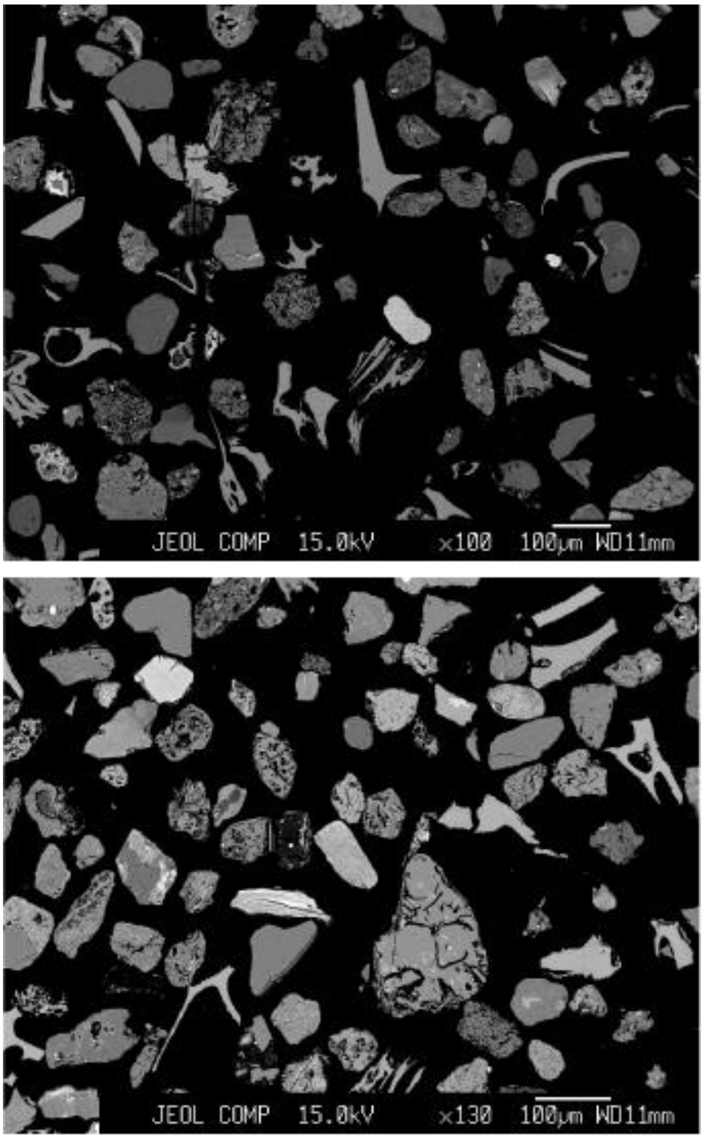

Figure S3. Scanning electron microscope (SEM) images of the Campanian Ignimbrite glass shards from the Vlasca site.

Table S2. Major oxide geochemical results from microprobe analyses of glass shards of the tephra layer (Campanian Ignimbrite/Y-5) at the Vlasca site. Data are presented as raw values. Analytical settings used for determining the glass-shard major oxides composition is presented at the Table S3.

**Sample**	**SiO_2**	**TiO_2**	**Al_2O_3**	**FeO**	**MnO**	**MgO**	**CaO**	**Na_2O**	**K_2O**	**P_2O_5**	**Cl-**	**Total**
STE1	61.07	0.38	18.17	2.88	0.09	0.55	2.17	4.06	8.04	0.061	0.346	97.81
STE2	60.43	0.40	18.54	2.75	0.18	0.33	1.68	6.3	6.67	0.021	0.729	98.02
STE3	61.01	0.44	18.65	2.84	0.23	0.40	1.62	6.5	7.47	0.010	0.741	99.91
STE4	61.23	0.33	18.11	2.68	0.12	0.47	1.98	4.41	8.3	0.103	0.409	98.13
STE5	61.55	0.37	18.23	2.92	0.11	0.58	2.22	4.54	7.98	0.133	0.367	99.00
STE6	60.49	0.45	18.4	2.76	0.25	0.36	1.67	6.03	7.88	0.024	0.722	99.03
STE7	60.82	0.37	18.67	2.84	0.19	0.32	1.73	6.35	7.43	0.039	0.718	99.48
STE8	60.53	0.43	18.58	2.92	0.20	0.34	1.7	6.27	7.34	0.059	0.711	99.08
STE9	60.77	0.45	18.57	2.8	0.26	0.31	1.64	6.55	7.17	0.044	0.785	99.35
STE10	61.37	0.45	18.8	2.85	0.21	0.34	1.65	6.53	6.8	0.033	0.692	99.73
STE11	61.3	0.44	18.61	2.82	0.25	0.35	1.62	6.69	7.28	0.047	0.748	100.16
STE12	61.19	0.48	18.71	2.86	0.29	0.29	1.62	6.77	6.85	0.025	0.708	99.79
STE13	60.84	0.44	18.48	2.89	0.22	0.35	1.67	5.9	8.12	0.054	0.708	99.66
STE14	60.86	0.43	18.66	2.93	0.22	0.36	1.72	6.65	6.84	0.108	0.726	99.51
STE15	61.18	0.43	18.7	2.91	0.24	0.33	1.79	5.91	7.67	0.016	0.710	99.88
STE16	60.77	0.37	18.73	2.73	0.23	0.31	1.64	6.61	6.75	0.056	0.739	98.93
STE17	60.42	0.43	18.44	2.89	0.18	0.34	1.62	6.56	6.85	0.066	0.726	98.53
STE18	60.73	0.35	18.2	3.4	0.13	0.71	2.52	3.49	9.2	0.151	0.328	99.21
STE19	60.91	0.40	18.56	2.84	0.17	0.35	1.73	6.28	7.12	0.051	0.727	99.15
STE20	61.25	0.41	18.58	2.87	0.21	0.33	1.7	6.69	7.09	0.046	0.727	99.90
STE21	60.54	0.45	18.57	2.84	0.23	0.37	1.65	6.52	6.96	0.042	0.740	98.92
STE22	59.22	0.38	18	3.45	0.11	0.79	2.67	3.08	9.46	0.110	0.291	97.56

STE23	59.84	0.38	18.55	2.83	0.24	0.32	1.59	6.65	6.71	0.044	0.743	97.89
STE24	62.09	0.33	18.47	2.69	0.13	0.48	1.94	5.03	7.92	0.078	0.457	99.62
STE25	60.6	0.43	18.39	2.88	0.23	0.35	1.58	6.31	6.69	0.034	0.739	98.24
STE26	61.43	0.36	18.45	3.04	0.11	0.58	2.18	5.12	7.55	0.068	0.339	99.22
STE27	62.22	0.38	18.54	2.94	0.13	0.59	2.16	3.43	7.19	0.110	0.346	98.04
STE28	60.36	0.39	18.68	2.8	0.23	0.35	1.66	6.52	6.9	0.069	0.720	98.67
STE29	60.73	0.42	18.55	2.81	0.20	0.31	1.62	6.61	6.95	0.051	0.733	98.98
STE30	61.05	0.40	18.75	2.89	0.24	0.35	1.6	6.8	7.35	0.029	0.742	100.19
STE31	59.84	0.38	18.08	2.62	0.21	0.37	1.66	5.58	7.19	0.093	0.459	96.47
STE32	60.4	0.42	18.46	2.83	0.22	0.32	1.62	6.2	6.82	0.014	0.738	98.05
STE33	60.46	0.45	18.45	2.89	0.18	0.29	1.71	6.63	7.02	0.069	0.695	98.84
STE34	60.45	0.43	18.39	2.79	0.21	0.35	1.8	6.39	6.96	0.091	0.685	98.55
STE35	61.03	0.39	18.31	2.92	0.23	0.36	1.65	6.51	7.09	0.054	0.735	99.28
STE36	60.58	0.45	18.35	2.91	0.23	0.30	1.72	6.66	6.87	0.046	0.703	98.82
STE37	61.71	0.38	18.53	2.69	0.17	0.39	1.66	5.92	7.53	0.017	0.527	99.53
STE38	61.67	0.38	18.41	2.75	0.15	0.51	2.06	4.53	8.27	0.076	0.378	99.18
STE39	60.79	0.44	18.61	2.8	0.24	0.36	1.72	6.38	7.32	0.017	0.711	99.39
STE40	60.86	0.41	18.44	2.81	0.26	0.30	1.75	6.67	7.25	0.008	0.697	99.46
STE41	61.2	0.39	18.7	2.84	0.21	0.34	1.92	5.85	7.14	0.005	0.751	99.35
STE43	60.9	0.41	18.62	2.8	0.23	0.32	1.68	6.22	7.43	0.064	0.696	99.37
STE44	60.31	0.42	18.44	2.86	0.21	0.29	1.61	6.6	6.8	0.059	0.728	98.33
Average	*60.86*	*0.41*	*18.49*	*2.86*	*0.20*	*0.39*	*1.79*	*5.94*	*7.35*	*0.06*	*0.64*	*98.98*
St. Dev.	*0.57*	*0.04*	*0.19*	*0.15*	*0.05*	*0.11*	*0.25*	*1.00*	*0.63*	*0.03*	*0.15*	*0.77*

Table S3. Analytical settings used for determining the glass-shard major oxides composition at Bayerisches GeoInstitut, Bayreuth University. Order of measuring elements (first to last): Na, Si, K, Ca, Fe, Mg, Al, P, Ti, Mn, Cl^{-}.

**Element**	**Standard (Standard-Block)**	**Measuring time (peak/bkgr.)**
Si /Kα	C-Forsterite (Cameca Block5)	30/15
Mn /Kα<br>Ti /Kα	C-$MnTiO_3$ (Cameca Oxides (2))	40/20<br>40/20
Al /Kα	C-Spinel (Cameca Block5)	30/15
Fe /Kα	Fe-GP40 (GP40)	30/15
Mg /Kα	C-Enstatite (Cameca Block5)	30/15
Ca /Kα	C-Wollastonite (Cameca Oxides (2))	30/15
K /Kα	C-Orthoclase (Cameca Oxides (2))	30/15
Na /Kα	C-Albite (Cameca Oxides (2))	10/5
P /Kα	C-Apatite (Cameca Oxides (2))	60/30
Cl^{-} /Kα	C-Vanadinite (Cameca Oxides (2))	30/15

4.2. Age model

Obtaining a reliable age model can be achieved in several ways. In loess research, the most common ones are by numerical dating (using luminescence[16–18] or radio carbon dating[19,20]) or by correlation to isotopic Greenland ice sheet $\delta^{18}O$ or speleothem records[21,22]. Establishing an age mode relying on luminescence dating has serious limitations in precision. Constructing such the age model is challenging, especially when using ages with 2-σ uncertainty. Since climate proxy parameters of the studied sections show a pacing comparable to millennial scale oscillation, using a correlative age model can significantly reduce the uncertainties. Such short scale millennial oscillations are reported in Southeastern European lacustrine records[23,24], including the Black Sea[25,26] (albeit Ménot and Bard[27] reported a lack in D/O interstadial signatures in the mean annual lake surface temperatures), and also in speleothem records representing Mediterranean climate[28,29]. Accordingly, recording millennial scale climatic oscillations in loess may be expected[22]. However, beside the correlative method, it is still not possible to relate fluctuations of loess proxies to certain interstadial-stadial events due to low resolution and lack in precision of dating techniques. Up to now, it has never been dated, and therefore convincingly demonstrated, that abrupt climate changes recorded in loess match individual D/O events. This problem is not only restricted to loess, but prevalent in many records[30]. Since the abrupt changes recorded in loess and D/O events might not coeval, using a correlative age model might increase bias to non-climatic forcing (instead of increasing precision). In particular the grain-size can be influenced by many factors beyond a straightforward relation to climate changes. The grain-size distribution strongly depends on the proximity to source area, sediment availability, environmental conditions at the source, during transport and deposition, trapping mechanisms, and even on the basic physical background of dust particle transport and deposition mechanisms[31]. Moreover, it has been demonstrated that grain-size records from adjacent sections (only few tens of meters apart) at

the Chinese Loess Plateau show different short term fluctuation, even if the long term patterns overlap[32]. Zeeden et al.[22] proposed frequency dependence of magnetic susceptibility (χ_{fd}) as a sensitive record for changes in sediment humidity and potentially the best proxy for indicating variations in D/O events scaling. However, here we show that albeit the general trends of the Urluia, Vlasca and Rasova[22] sections are quite similar (Fig. S6), there are also notable differences in the amplitude of fluctuations and short term patterns. This indicates an important influence of local conditions to the extent of χ_{fd} oscillation, and therefore again might record a bias to changes not directly related to climate change in the wider region.

To obtain the age model in our study, we did not fully rely on the correlative age model or luminescence dating. Since we cannot certainly claim that D/O events are coeval with fluctuations in loess records, correlating our records to D/O events might increase a bias rather than precision of the age model. We compiled an age model that reduces the tie points to a minimum, and uses only the most certain tie points. In this way, the resolution of the age model decreases, but also the potential bias to error is minimized. We use the Campanian Ignimbrite tephra as a robust tie point at both sections, and only the correlations that are strongly supported by luminescence dating are used as tie points. Since the Vlasca section was not directly dated so far, this section is correlated to the general trends of proxy parameters at the Urluia section. We feel certain about this approach because both sections indicate very similar patterns in grain-size and environmental magnetic data. Details on the age models from both sections are presented in following chapters.

4.2.1. Age model based on luminescence dating

In order to obtain an age-depth model for the loess sequence at Urluia, seven luminescence samples are used in a first step (Fig. S2). The thorough investigation of the luminescence samples shows no problematic behavior. This demonstrates the general suitability of the $pIRIR_{290}$ protocol for these samples. The $pIRIR_{290}$ signal is argued to be not affected by fading[3,4], which was supported by our fading measurements resulting in a negative mean g-value. Since the measured fading rates are likely to be a laboratory artifact[33], equivalent doses were not corrected for fading. The resulting ages increase constantly with depth. The layer of the Campanian Ignimbrite tephra dated to 39.93 ± 0.1 ka[2,14,34] presents a robust tie point and it is used as an independent test for the precision of the luminescence ages. The luminescence sample C-L3709 at 6.0 m, ~10 cm above the tephra layer, indicates an age of 44.1 ± 3.4 ka. Using the lower boundary of 1 σ uncertainty (Fig. S2), the time period shows slightly older age for the sediment deposited after the Campanian Ignimbrite tephra accumulation. The age is ~2-3 ka older than expected, and even if this does not indicate a perfect match, the ages might be assumed as reliable indications of the sedimentation timeframe on a more general scale, especially when considering 2-σ uncertainty (under which the ages overlap).

4.2.2. Correlative age model

It is challenging to compile the age model using luminescence ages with 2 σ uncertainty, since the uncertainty is considerably high (up to ~16.2 ka years for the C-L3708 sample and 2-σ). To reduce the uncertainty we compile a correlative age model based on the correlation of proxy data from Urluia to millennial scale palaeoclimatic patterns observed in $\delta^{18}O$ data from the Greenland ice sheet[35] (Fig. S4, Table S4). For the correlation to the Greenland $\delta^{18}O$ record we used the frequency dependent magnetic susceptibility (χ_{fd}) data as suggested by Zeeden et al.[22], but also paid attention to the patterns in grain-size distribution. Luminescence ages then cross-checked this model (Fig. S2). Obtaining an age-depth model for Vlasca

(Fig.S5 and Table S4) was more challenging since the numerical time control of the luminescence dating is not given. However, the timing of the deposited Campanian Ignimbrite tephra layer allows a straightforward correlation to the Urluia record. The similar patterns in the χ_{fd} and especially grain-size from Vlasca and Urluia allow a convincing correlation of these two sections, and thus, an indirect correlation of the Vlasca sequence to the Greenland $\delta^{18}O$ record. Furthermore, the Vlasca and Urluia records were compared with the close-by Rasova section providing an age-depth model[22], supporting and additionally verifying the presented age models (Fig. S6).

The onset of the studied part of the Urluia section is tentatively correlated to Greenland Stadial (GS) 15 (before 55.4 ka; Fig. S4). Following an increase in χ_{fd}, high values at 8.06 m are corresponding to Greenland Interstadial (GI) 14. The following points of correlation are associated with increases in χ_{fd} at 7.58 m, 7.18 m, and 6.70 m, and are related to GI 13, GI 12, and GI 11 (Fig. S4), respectively. The luminescence sample C-L3708 at 6.90 m indicates a timing of deposition of this layer is 54.2+/-4.1 with 1-σ uncertainty, or 54.2+/-8.0 with 2-σ uncertainty (Fig. S2). The correlative age (~45 ka for 6.90 m) is in the lower range of the 2-σ uncertainty. However, as noted above, it is possible that luminescence ages tend to show an age in a lower range of the 2-σ uncertainty.

Establishing the onset of the Vlasca profile is very challenging since the grain-size is heavily influenced by high sand content that is very likely related to a local flooding event and therefore regarded as a non-climatic signal. The base of the section is deposited as a sandy layer. Generally high χ_{fd} and sand content in these layers (Fig. 2) indicate that such grain-size distribution is not a climatic signal, but rather related to the increased fluvial dynamics or flooding of the Danube River that supplied the Danube banks with high amounts of sand and coarse particles suitable for aeolian transport. We related the onset of the Vlasca section to GS 12, while the increases in χ_{fd} at 10.08 m and 9.60 m are related to GI 11 and GI 10 (Fig. S5).

However, such correlation for the older part of Vlasca section is highly debatable, and should be treated with special caution.

The preserved tephra layer is a safe chronological marker and it is used as a reliable tie point at both sections with an age of 39.93 ± 0.1 ka[2,14,34]. Above the tephra layer, χ_{fd} and fine grain-size fractions at both sections indicate a prominent peak in data, which we relate to Greenland Interstadial (GI) 8 (Figs. S4 and S5). At Urluia, the luminescence sample is present in this depth interval (Fig. S2). As mentioned above, it exhibits an age of 44.1 ± 3.4 ka (with 1-σ uncertainty), a clearly older age than the deposition of Campanian Ignimbrite tephra. However, the timing of the GI 8 at 38,300 years ago is in the range of the luminescence age uncertainty. The enhanced peak in χ_{fd} and finer grain-size fraction are also observed at Vlasca, and this layer is related to GI 8 as well.

At Vlasca, three prominent peaks in χ_{fd} and finer grain-size fraction stands out at ~8.2 m, ~7.86 m and ~7.52 m (Fig. S5). These layers were correlated to GI 7, GI 6 and GI 5. At Urluia, only two clear peaks in the fine fractions can be observed. Two luminescence ages at 5.20 m and 4.50 m support the correlation of sediment interval from 4-6 m to tentatively span the timeframe of the past 30,000 to 40,000 years (Fig. S2). The luminescence age at 4.5 m (32.5 ± 2.5 ka; C-L3712) suggests a correlation of the younger peak in grain-size data to the GI 5. However, the luminescence age of 36.2 ± 2.8 ka (C-L3711) cannot clearly specify the increase in fine fractions to GI 6 or GI 7. We argue that this increase in finer fractions corresponds to GI 6, while GI 7 is recorded at a depth of 5.62 m. Two main criteria for such correlation are 1) the general tendency of the here presented luminescence ages to indicate slightly older ages or expected ages to be in the younger uncertainty limit and 2) observed similarities to the Vlasca section. Regarding the first criterion, the luminescence age that dates the sediment just below the observed increase in fine particles would support a correlation with 1-σ uncertainty of this layer to both, GI 6 and GI 7. However, considering that the means

of the older luminescence ages are slightly overestimated, especially indicated by the Campanian Ignimbrite, it is more likely that the lower limits of the ages uncertaintiesgives a better assumption of the 'true' age. Therefore, we correlated the peak of higher contribution of fine particles at 5.02 m to GI 6, while the relatively weaker increase at 5.62 m in χ_{fd} and grain-size we relate to GI 7 (Fig. S4). Generally higher χ_{fd} and slightly finer particles at 5.62 m than at 5.02 m are in better agreement with the values related to GI 7 at Vlasca.

Establishing the following two tie points of the presented age model was generally straightforward, since the χ_{fd} indicate a notable increase in the values, while the U-ratio indicate a decrease. At Urluia, the layers at 3.60 m and 3.32 m are related to GI 4 and GI 3, while at Vlasca these interstadials are related to depths of 6.50 m and 6.22 m (Figs. S4 and S5). However, although the χ_{fd} indicates a clear increase in humidity, the grain-size distribution shows a higher contribution of coarser fractions (Figs. S4 and S5). Therefore, it may be debated if this layer showing a high contribution of coarse particles at both sections corresponds to Heinrich event (HE) 3. Nonetheless, we argue that only part of the observed increase in grain-size is related to HE 3, where the coarser particles are a consequence of increased winter severity as recorded in the Black Sea record[25] that covers GS 5 and 4 (Fig. S7). In addition, the luminescence age at 3.3 m (C-L3713) supports the established correlation (Fig. S2).

An increase in χ_{fd} and finer grain-size at 5.16 m at the Vlasca section is correlated to GI 2 (Fig. S5). Although similar increase in χ_{fd} cannot be observed at Urluia, but a clearly similar pattern in grain-size decrease is observed at 2.68 m and correlated to GI 2 (Fig. S4). Nevertheless, such correlation is not strongly supported by luminescence age at 2 m (25.0±1.9 ka, C-L3715). The youngest uncertainty of this age is ~23 ka, indicating the timing of the GI 2 somewhere above 2 m at Urluia. However, a correlation based on this luminescence age would indicate very low sedimentation rates for last 23 ka, which includes most of the Last

Glacial Maximum. Although we cannot rule this scenario out, we assume it as unlikely. Therefore, we correlate the increase in grain-size fractions and slight decrease in χ_{fd} at 2.30 m to GS 3. Based on the relation of χ_{fd} and grain-size from Urluia and Vlasca, we related the interval at 4.02 m at Vlasca to GS 3 (Figs. S4 and S5).

The luminescence age suggests for the sediment at 1.5 m depth a lower uncertainty age of ~19.5 ka (Fig. S2). According to that age, the sediment above showinga decrease in the <5 µm fractions and χ_{fd} at 1.30 m is related to HE 1. At Vlasca, HE 1 is correlated to the depth of 2.54 m. Such correlation is in good agreement with a correlation obtained at the Rasova section[22], where the HE 1 is recorded shortly after three prominent peaks in χ that can be also observed at the here discussed sections (Fig. S6).

For the timing of the Younger Dryas we related a decrease in χ_{fd} and an increase in grain-size fractions after the second prominent peak in those proxies observed in the younger part of sectionsto the Younger Dryas. We argue that those lower values are generally unlikely to represent the Holocene climate. A following increase in χ_{fd} and fine grain-size is related to the onset of the Holocene. The same solution was obtained for the Rasova section[22], which makes a regional correlation more coherent (Fig. S6).

Table S4. Tuning points of the Urluia and Vlasca sections correlation to $\delta^{18}O$ from Greenland ice sheet [35]

Profile	Tuning point	Depth (m)	Age (years)
Urluia	GS 15	8.48	55,400
Urluia	GI 14	8.06	54,800
Urluia	GI 13	7.58	49,800
Urluia	GI 12	7.18	47,350
Urluia	GI 11	6.70	43,400
Urluia	Campanian Ignimbrite tephra	6.22	39,930
Urluia	GI 8	5.90	38,300
Urluia	GI 7	5.62	35,300
Urluia	GI 6	5.02	33,500
Urluia	GI 5	4.38	32,250
Urluia	GI 4	3.60	28,500
Urluia	GI 3	3.32	27.400
Urluia	GI 2	2.68	22,700
Urluia	GS 3	2.30	20,450
Urluia	HE 1	1.46	15,450
Urluia	Holocene transition	0.88	11,500
Vlasca	GS 12	10.72	44,000
Vlasca	GI 11	10.06	43,400
Vlasca	GI 10	9.60	41,750
Vlasca	Campanian Ignimbrite tephra	9.12	39,930
Vlasca	GI 8	8.58	38,300
Vlasca	GI 7	8.20	35,300
Vlasca	GI 6	7.86	33,500
Vlasca	GI 5	7.52	32,250
Vlasca	GI 4	6.50	28,500
Vlasca	GI 3	6.20	27.400
Vlasca	GI 2	5.16	22,700
Vlasca	GS 3	4.02	20,450
Vlasca	HE 1	2.54	15,450
Vlasca	Holocene transition	1.52	11,500

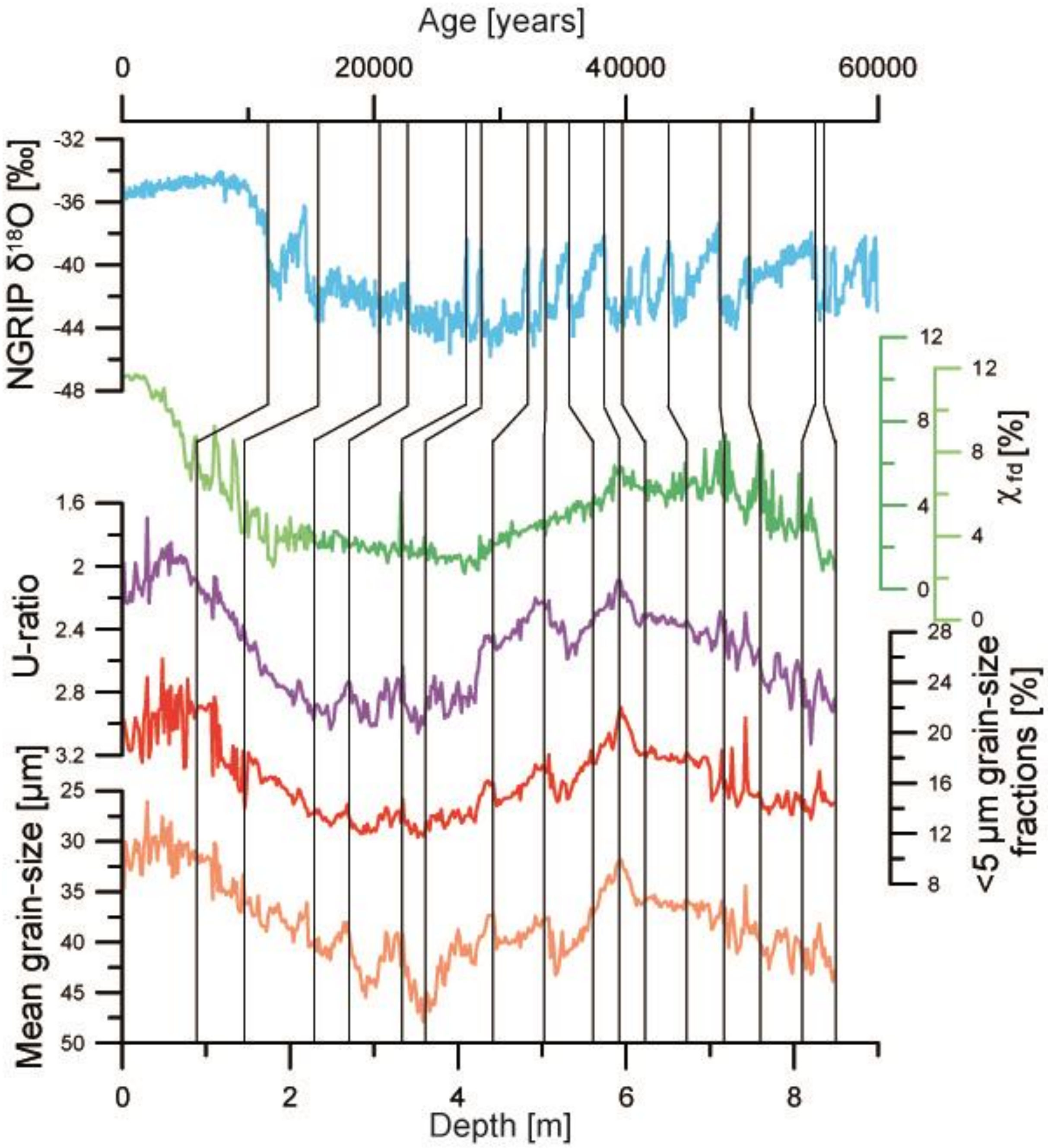

Figure S4. Correlation between the $\delta^{18}O$ data from Greenland ice sheet[35] (blue line) and proxies from the Urluia section (green lines for χ_{fd} (see the discussion in Results chapter in main manuscript for the explanation of two different scales), purple line for U-ratio, red line for fine particles (<5 µm) and orange line represents mean grain-size).

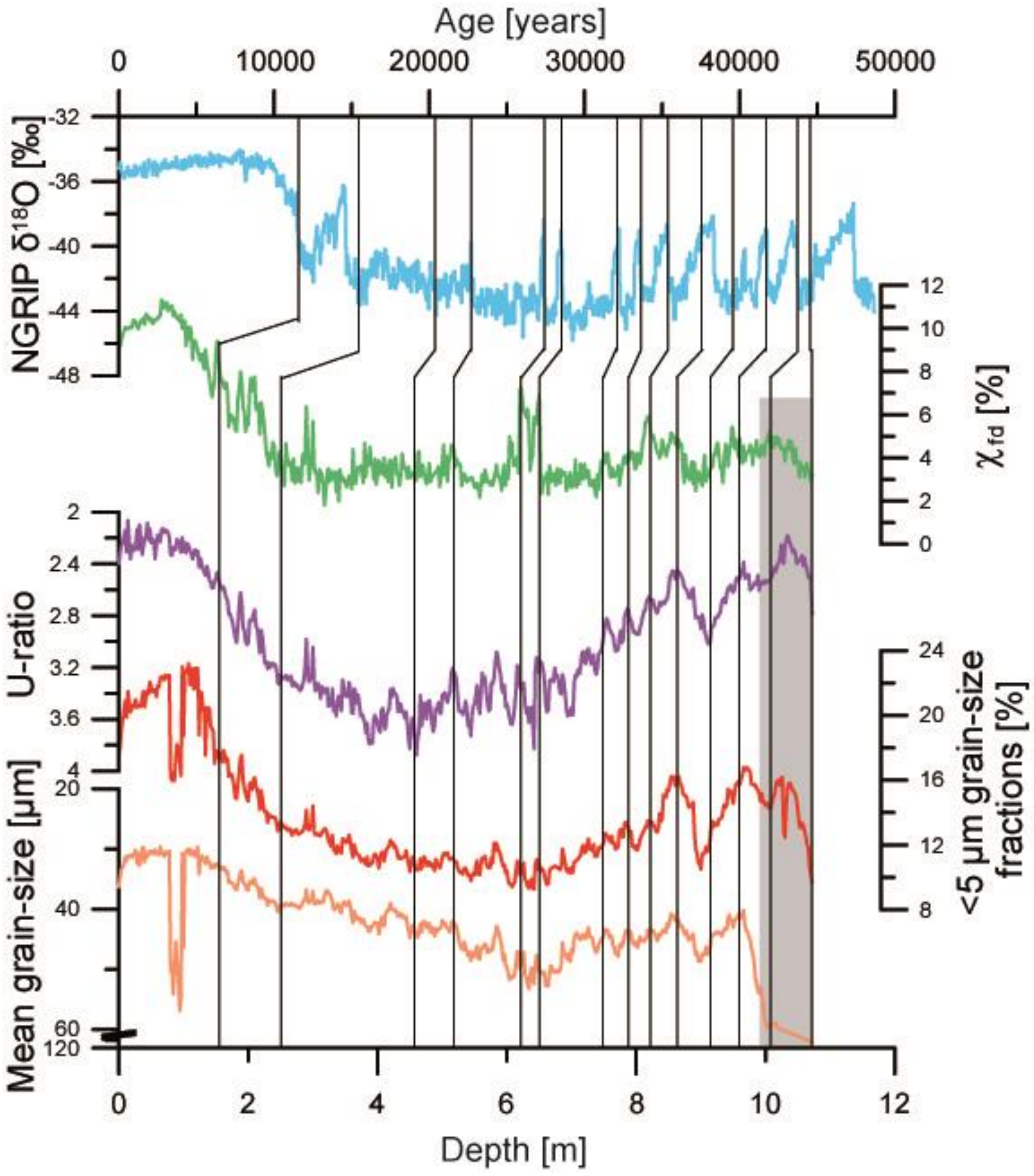

Figure S5. Correlation between the $\delta^{18}O$ data from Greenland ice sheet[35] (blue line) and proxies from the Vlasca section (green line for χ_{fd}, purple line for U-ratio, red line for fine particles (<5 µm) and orange line for mean grain-size). Grey rectangle represents a sandy layer that is not considered for palaeoclimate reconstruction (not shown elsewhere).

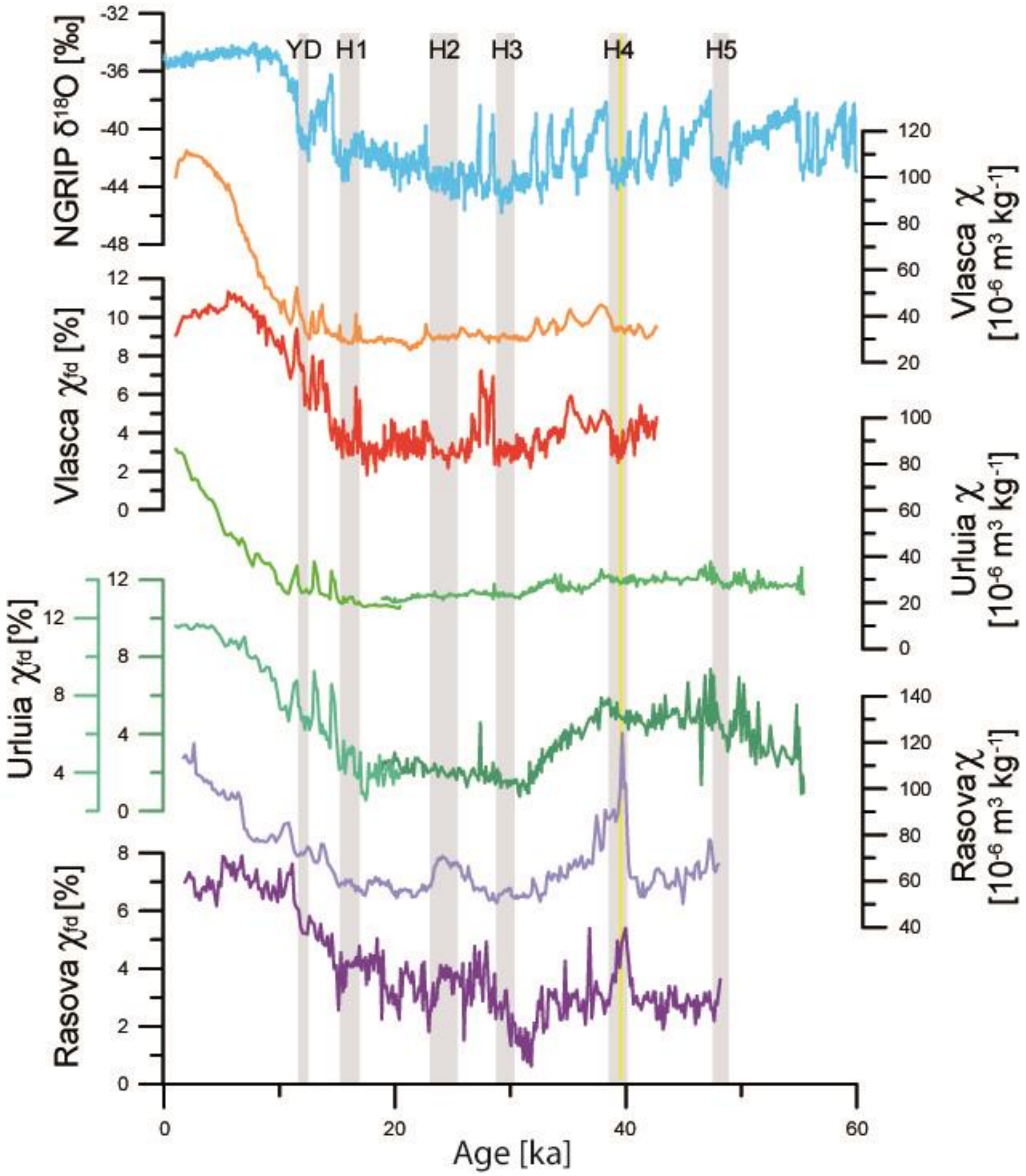

Figure S6. Correlation between the $\delta^{18}O$ data from Greenland ice sheet[35] (blue line), and χ and χ_{fd} from Vlasca (orange and red lines), Urluia (lines in different shades of green; (see the discussion in Results chapter in main manuscript for the explanation of two different scales) and Rasova[22] (lines in different shades of purple) sections presented on the correlative age models. Grey rectangles represent Heinrich Events, straight yellow line represent the timing of the Campanian Ignimbrite tephra deposition.

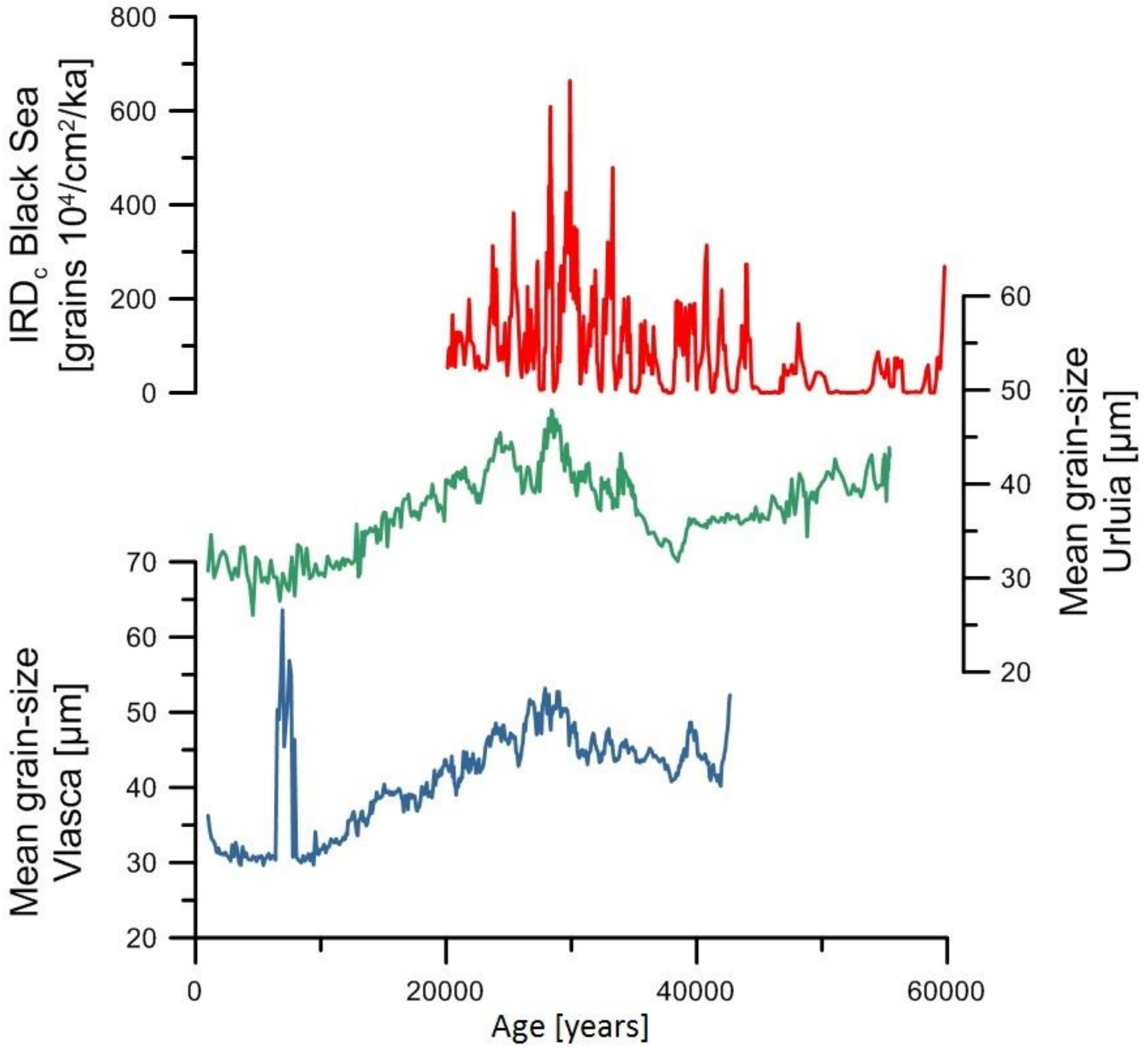

Figure S7. Comparison of mean grain-size from Vlasca (blue line) and Urluia (green line), and the accumulation rate of coastal ice-rafted detritus (IRD_C) from the Black Sea as an indication of winter severity[25,36].

4.2.3. Final age model

To obtain the final age model we have combined a correlative model and luminescence ages and used the most certain tie points only. The Campanian Ignimbrite tephra is used as a robust tie point at both sections. The following two tie points at Urluia are at 4.5 m (32.5 ka) and 3.3 m (28.7 ka), where luminescence samples show very good relation to the correlative age model (Fig. S2). The next tie point is at 0.9 m (11.5 ka), where an increase in all proxies indicates the onset of the Holocene. Since the Vlasca section has no further time control, we relate obvious changes in χ_{fd} which can be observed at both sections to obtain the same age. The low value in χ_{fd} at 7.38 m at Vlasca is related to the low value at 4.08 m at Urluia and assumed to have an age of 31,170 years (Figs. 2, S4 and S5). An increase in χ_{fd} at 6.22 m is related to an increase at 3.32 m at Urluia, and has an assumed age of ~28,760 years (Figs. 2, S4 and S5). An increase in χ_{fd} at 2.1 m and 1.52 m at Vlasca are related to increases at 1.32 m (~14,630 years) and 0.88 m (11.500 years) at Urluia (Figs. 2, S4 and S5). Table S5 shows tie points for both sections.

Finally, it is important to state that no matter which age model is used, the conclusions presented in this study are valid for all three age models (Fig. S8). The difference to data from the Middle Danube Basin is also evident when no age model additional to the Campanian Ignimbrite occurrence is applied.

Table S5. Tie points of the final age models for the Urluia and Vlasca sections.

Profile	Depth [m]	Age [years]
Urluia	8.48	55,400
Urluia	6.22	39,930
Urluia	4.50	32,500
Urluia	3.30	28,700
Urluia	0.88	11,500
Vlasca	10.00	43,000
Vlasca	9.12	39,930
Vlasca	7.38	31,170
Vlasca	6.20	28,760
Vlasca	2.10	14,630
Vlasca	1.52	11,500

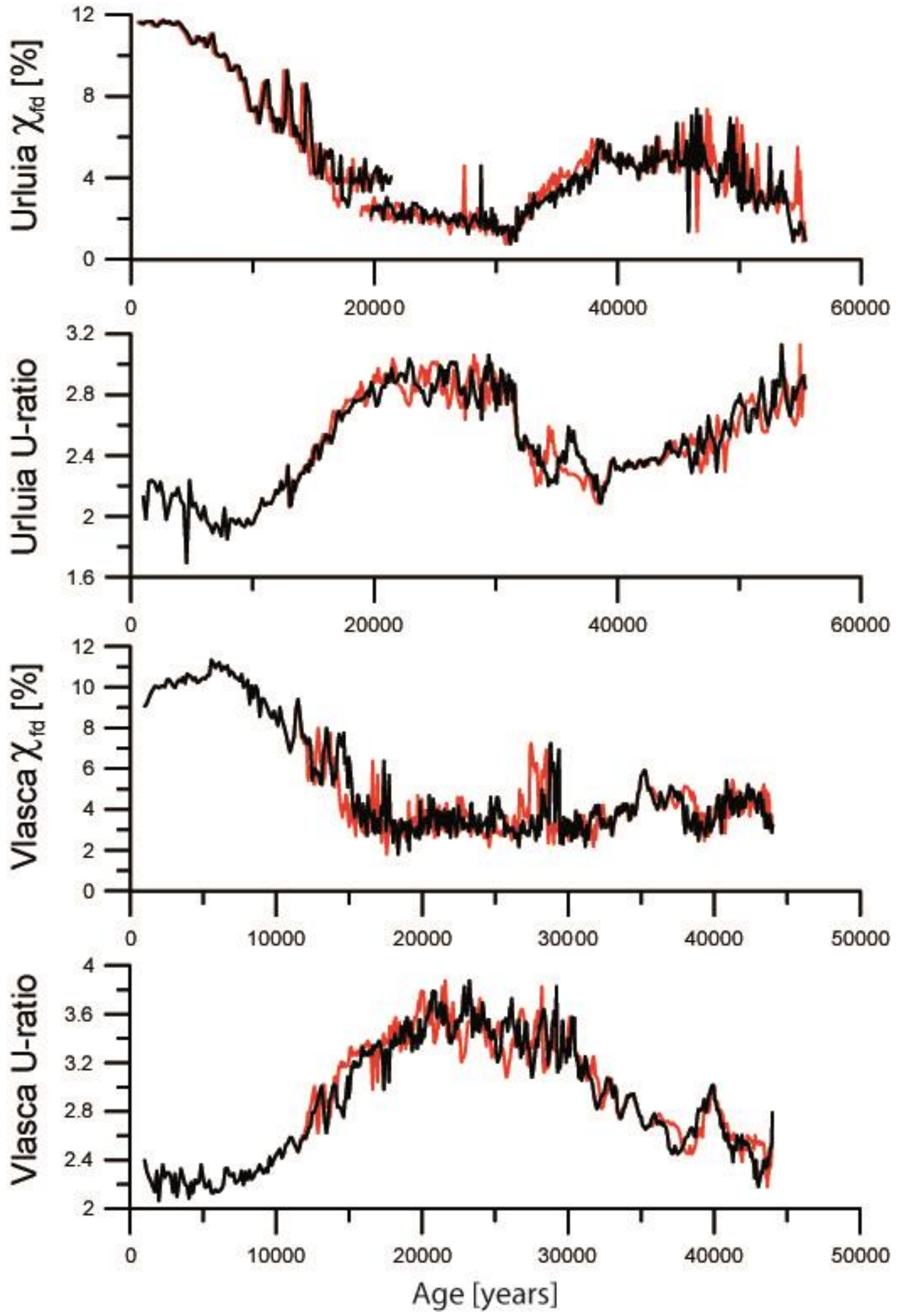

Figure S8. Comparison of the correlative age model (red lines) and final age model (black lines). Note the same general trends regardless which age model is used.

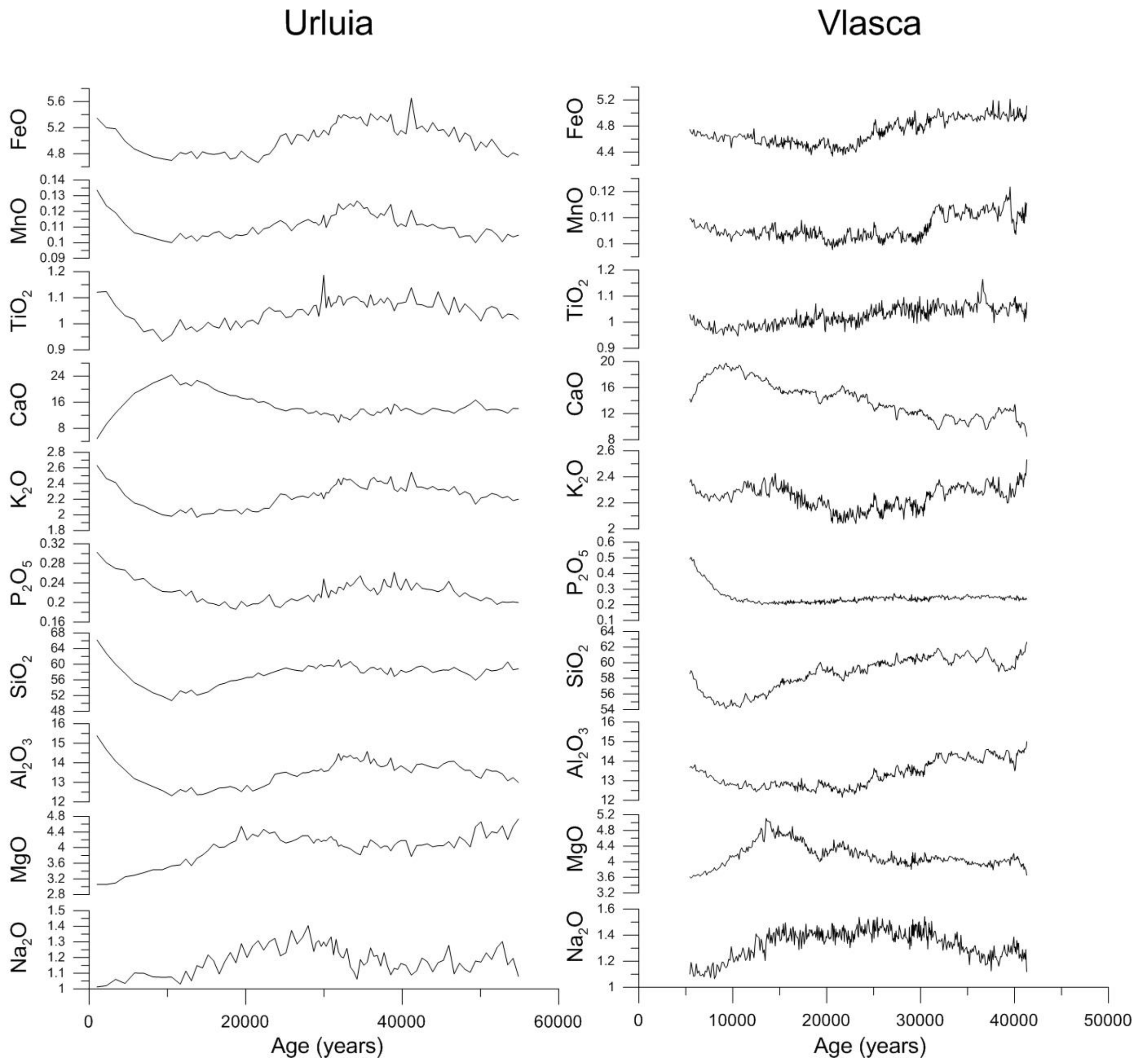

Figure S9. Major elements from the Urluia and Vlasca sections. Major elements are presented in %.

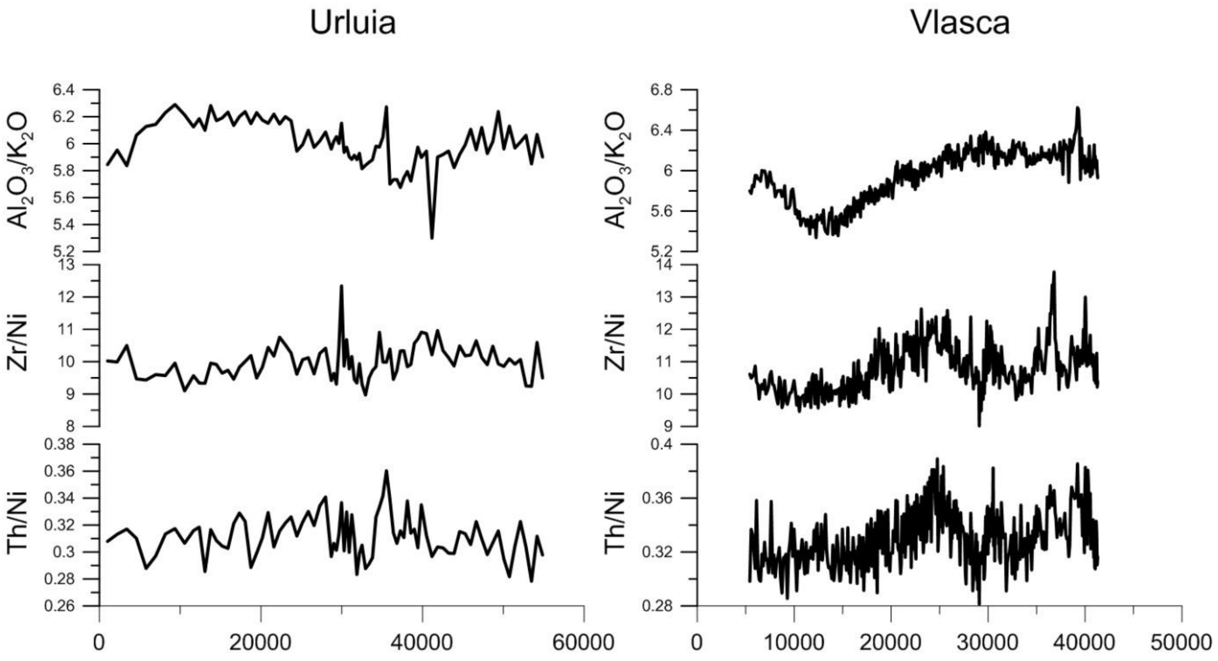

Figure S10. Geochemical ratios commonly used to indicate possible changes in the provenance. No ratio indicates major change in provenance, only Al2O3/K2O ratio at Vlasca suggests a slight increase in K-feldspars between 20-10 ka.

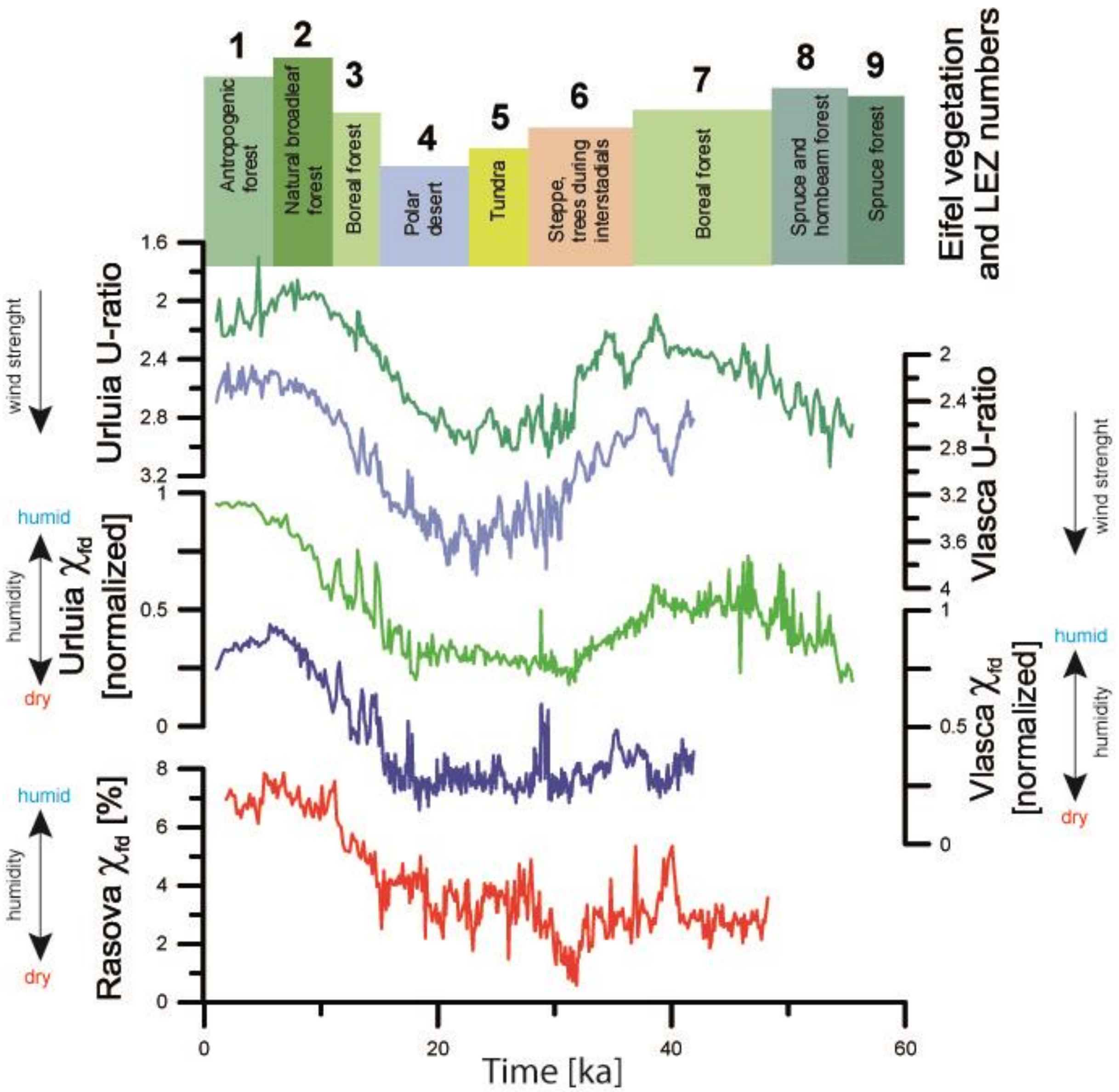

Figure S11. Frequency dependent magnetic susceptibility from Rasova[22] (red line), Vlasca (blue line) and Urluia (green line), U-ratio from Vlasca (pale blue line) and Urluia (dark green line) compared to the "Landscape Evolution Zones" (LEZ) established from Eifel maar sediments[37] of the last 60,000 years and generally valid for central Western Europe. Note the similar timing of the transition of LEZ 7 (boreal forest) to 6 (steppe) and the continentalization of the Lower Danube Basin.

Supplementary references

1. Fitzsimmons, K. E. & Hambach, U. Loess accumulation during the last glacial maximum: Evidence from Urluia, southeastern Romania. *Quat. Int.* **334–335,** 74–85 (2014).
2. Fitzsimmons, K. E., Hambach, U., Veres, D. & Iovita, R. The Campanian Ignimbrite Eruption: New Data on Volcanic Ash Dispersal and Its Potential Impact on Human Evolution. *PLoS ONE* **8,** e65839 (2013).
3. Buylaert, J.-P. *et al.* A robust feldspar luminescence dating method for Middle and Late Pleistocene sediments: Feldspar luminescence dating of Middle and Late Pleistocene sediments. *Boreas* **41,** 435–451 (2012).
4. Thiel, C. *et al.* Luminescence dating of the Stratzing loess profile (Austria) – Testing the potential of an elevated temperature post-IR IRSL protocol. *Quat. Int.* **234,** 23–31 (2011).
5. Galbraith, R. F., Roberts, R. G., Laslett, G. M., Yoshida, H. & Olley, J. M. Optical Dating of Single and Multiple Grains of Quartz from Jinmium Rock Shelter, Northern Australia: Part I, Experimental Design and Statistical Models*. *Archaeometry* **41,** 339–364 (1999).
6. Durcan, J. A., King, G. E. & Duller, G. A. T. DRAC: Dose Rate and Age Calculator for trapped charge dating. *Quat. Geochronol.* **28,** 54–61 (2015).
7. Liritzis, I., Stamoulis, K., Papachristodoulou, C. & Ioannides, K. A re-evaluation of radiation dose-rate conversion factors. *Mediterr Archaeol Archaeom* **13,** 1–15 (2013).
8. Huntley, D. J. & Baril, M. R. The K content of the K-feldspars being measured in optical dating or in thermoluminescence dating. *Anc. TL* **15,** 11–12 (1997).
9. Huntley, D. J. & Hancock, R. G. V. The Rb contents of the K-feldspar grains being measured in optical dating. *Anc. TL* **19,** 43–46 (2001).
10. Bell, W. T. Alpha dose attenuation in quartz grains for thermoluminescence dating. *Anc. TL* **12,** 4–8 (1980).

11. Guérin, G., Mercier, N., Nathan, R., Adamiec, G. & Lefrais, Y. On the use of the infinite matrix assumption and associated concepts: A critical review. *Radiat. Meas.* **47,** 778–785 (2012).
12. Prescott, J. R. & Hutton, J. T. Cosmic ray contributions to dose rates for luminescence and ESR dating: Large depths and long-term time variations. *Radiat. Meas.* **23,** 497–500 (1994).
13. Kreutzer, S., Schmidt, C., DeWitt, R. & Fuchs, M. The a-value of polymineral fine grain samples measured with the post-IR IRSL protocol. *Radiat. Meas.* **69,** 18–29 (2014).
14. Veres, D. *et al.* The Campanian Ignimbrite/Y5 tephra layer – A regional stratigraphic marker for Isotope Stage 3 deposits in the Lower Danube region, Romania. *Quat. Int.* **293,** 22–33 (2013).
15. Scaillet, S., Vita-Scaillet, G. & Rotolo, S. G. Millennial-scale phase relationships between ice-core and Mediterranean marine records: insights from high-precision 40Ar/39Ar dating of the Green Tuff of Pantelleria, Sicily Strait. *Quat. Sci. Rev.* **78,** 141–154 (2013).
16. Marković, S. B. *et al.* Environmental dynamics and luminescence chronology from the Orlovat loess–palaeosol sequence (Vojvodina, northern Serbia). *J. Quat. Sci.* **29,** 189–199 (2014).
17. Stevens, T., Marković, S. B., Zech, M., Hambach, U. & Sümegi, P. Dust deposition and climate in the Carpathian Basin over an independently dated last glacial–interglacial cycle. *Quat. Sci. Rev.* **30,** 662–681 (2011).
18. Timar, A. *et al.* Optical dating of Romanian loess using fine-grained quartz. *Quat. Geochronol.* **5,** 143–148 (2010).
19. Újvári, G. *et al.* AMS 14C and OSL/IRSL dating of the Dunaszekcső loess sequence (Hungary): chronology for 20 to 150 ka and implications for establishing reliable age–depth models for the last 40 ka. *Quat. Sci. Rev.* **106,** 140–154 (2014).

20. Hatté, C. *et al.* Excursions to C4 vegetation recorded in the Upper Pleistocene loess of Surduk (Northern Serbia): an organic isotope geochemistry study. *Clim Past* **9,** 1001–1014 (2013).
21. Yang, S. & Ding, Z. A 249 kyr stack of eight loess grain size records from northern China documenting millennial-scale climate variability. *Geochem. Geophys. Geosystems* **15,** 798–814 (2014).
22. Zeeden, C. *et al.* Millennial scale climate oscillations recorded in the Lower Danube loess over the last glacial period. *Palaeogeogr. Palaeoclimatol. Palaeoecol.* doi:10.1016/j.palaeo.2016.12.029
23. Panagiotopoulos, K., Böhm, A., Leng, M. J., Wagner, B. & Schäbitz, F. Climate variability over the last 92 ka in SW Balkans from analysis of sediments from Lake Prespa. *Clim Past* **10,** 643–660 (2014).
24. Feurdean, A. *et al.* Climate variability and associated vegetation response throughout Central and Eastern Europe (CEE) between 60 and 8 ka. *Quat. Sci. Rev.* **106,** 206–224 (2014).
25. Wegwerth, A. *et al.* Black Sea temperature response to glacial millennial-scale climate variability. *Geophys. Res. Lett.* **42,** 2015GL065499 (2015).
26. Shumilovskikh, L. S. *et al.* Orbital- and millennial-scale environmental changes between 64 and 20 ka BP recorded in Black Sea sediments. *Clim Past* **10,** 939–954 (2014).
27. Ménot, G. & Bard, E. A precise search for drastic temperature shifts of the past 40,000 years in southeastern Europe. *Paleoceanography* **27,** PA2210 (2012).
28. Fleitmann, D. *et al.* Timing and climatic impact of Greenland interstadials recorded in stalagmites from northern Turkey. *Geophys. Res. Lett.* **36,** L19707 (2009).
29. Ünal-İmer, E. *et al.* An 80 kyr-long continuous speleothem record from Dim Cave, SW Turkey with paleoclimatic implications for the Eastern Mediterranean. *Sci. Rep.* **5,** 13560 (2015).

30. Wunsch, C. Abrupt climate change: An alternative view. *Quat. Res.* **65,** 191–203 (2006).
31. Újvári, G., Kok, J. F., Varga, G. & Kovács, J. The physics of wind-blown loess: Implications for grain size proxy interpretations in Quaternary paleoclimate studies. *Earth-Sci. Rev.* **154,** 247–278 (2016).
32. Stevens, T. & Lu, H. Optically stimulated luminescence dating as a tool for calculating sedimentation rates in Chinese loess: comparisons with grain-size records. *Sedimentology* **56,** 911–934 (2009).
33. Buylaert, J.-P. *et al.* IRSL and post-IR IRSL residual doses recorded in modern dust samples from the Chinese Loess Plateau. *Geochronometria* **38,** 432–440 (2011).
34. Lowe, J. *et al.* Volcanic ash layers illuminate the resilience of Neanderthals and early modern humans to natural hazards. *Proc. Natl. Acad. Sci.* **109,** 13532–13537 (2012).
35. Andersen, K. K. *et al.* High-resolution record of Northern Hemisphere climate extending into the last interglacial period. *Nature* **431,** 147–151 (2004).
36. Wegwerth, A. *et al.* IRDc and accumulation rates of IRDc on sediment core M72/5_628-1. (2015).
37. Sirocko, F. *et al.* The ELSA-Vegetation-Stack: Reconstruction of Landscape Evolution Zones (LEZ) from laminated Eifel maar sediments of the last 60,000 years. *Glob. Planet. Change* **142,** 108–135 (2016).
